# Global Expression Profiling of Low Temperature Induced Genes in the Chilling Tolerant Japonica Rice Jumli Marshi

**DOI:** 10.1371/journal.pone.0081729

**Published:** 2013-12-12

**Authors:** Aakash Chawade, Angelica Lindlöf, Björn Olsson, Olof Olsson

**Affiliations:** 1 CropTailor AB, Lund, Sweden; 2 Systems Biology Research Centre, School of Life Sciences, University of Skövde, Skövde, Sweden; 3 Department of Pure and Applied Biochemistry, Lund University, Lund, Sweden; University of Delhi South Campus, India

## Abstract

Low temperature is a key factor that limits growth and productivity of many important agronomical crops worldwide. Rice *(Oryza sativa* L.) is negatively affected already at temperatures below +10°C and is therefore denoted as chilling sensitive. However, chilling tolerant rice cultivars exist and can be commercially cultivated at altitudes up to 3,050 meters with temperatures reaching as low as +4°C. In this work, the global transcriptional response to cold stress (+4°C) was studied in the Nepalese highland variety Jumli Marshi (spp. *japonica*) and 4,636 genes were identified as significantly differentially expressed within 24 hours of cold stress. Comparison with previously published microarray data from one chilling tolerant and two sensitive rice cultivars identified 182 genes differentially expressed (DE) upon cold stress in all four rice cultivars and 511 genes DE only in the chilling tolerant rice. Promoter analysis of the 182 genes suggests a complex cross-talk between ABRE and CBF regulons. Promoter analysis of the 511 genes identified over-represented ABRE motifs but not DRE motifs, suggesting a role for ABA signaling in cold tolerance. Moreover, 2,101 genes were DE in Jumli Marshi alone. By chromosomal localization analysis, 473 of these cold responsive genes were located within 13 different QTLs previously identified as cold associated.

## Introduction

The inherent ability of plants to endure low temperatures affects both their geographical distribution and overall productivity. Many cereal crops from temperate regions, e.g., wheat, barley, rye and oat, have evolved efficient protection mechanisms to tolerate freezing [1]. This tolerance is acquired through a process known as cold acclimation, which occurs at low, but above-zero temperatures [2]. On the other hand, the highly important staple food, rice, like many other tropical plants, is chilling sensitive and does not survive freezing temperatures even after cold acclimation. However, chilling susceptibility varies substantially among cultivars of the same species [3], indicating that even chilling sensitive crops, at least to a certain degree, can acclimate to cooler temperatures. For example, *japonica* rice cultivars show a greater tolerance to chilling than *indica* cultivars, although variations within *japonica* exist [4].

Rice is mainly grown in warm climates (>25°C), but exposure to low temperature (LT) is common for rice cultivated in temperate zones or at high elevations in several regions of Europe, South Asia and Southeast Asia. It is, therefore, of outstanding scientific and economic interest to understand chilling tolerance, identify key regulatory components of chilling acclimation, resolve mechanistic differences between tolerant and sensitive rice and apply this knowledge in the development of new, more tolerant rice cultivars.

It is known that cold acclimation leads to physiological and metabolic changes in cell and tissue structures as a result of an extensive reprogramming in gene expression [2], [5], [6], [7]. A large number of genes that are differentially expressed during cold acclimation have been identified and characterized in important cold hardy cash crops like wheat (*Triticum aestivum*) [8], barley (*Hordeum vulgare*) [9], [10] and oat (*Avena sativa*) [7], [11]. However, the plant species most extensively studied during cold stress and acclimation is a non-crop plant, *Arabidopsis thaliana*, where global transcriptional profiling experiments have identified several cold responsive genes [12], [13], [14], [15], [16], [17]. The complexity of genetic re-programming upon cold stress has also been demonstrated by various bioinformatics approaches [15], [18], [19], [20], [21].

Previously, Rabbani *et al.* [22] used a rice cDNA microarray of 1,718 ESTs and identified 36 cold responsive genes in two-week old seedlings of chilling tolerant rice Nipponbare (spp. *japonica*) exposed to +4°C for 24 hours. Cheng *et al.* [23] used a rice cDNA microarray of 5,855 unique ESTs and identified 121 cold responsive genes in 10 days old seedlings of the chilling tolerant rice CT6748-8-CA-17 (spp. *japonica*) treated at +10°C for up to 24 hours. In another study, Oda *et al*. [24] used the 44K Agilent oligonucleotide microarray to compare two *japonica* cultivars Sasanishiki and Hitomebore exposed to low temperature stress (19°C) at the reproductive stage. Microarray analysis of anthers from the two cultivars led to the identification of 356 differentially expressed genes in either or both cultivars. Yun *et al.* [25] analyzed genes induced by chilling stress (+10°C) in Nipponbare using microarrays representing 40,000 genes and identified 8,668 differentially expressed genes. Mittal et al. [26] performed microarray analysis of the *indica* rice Pusa Basmati that was cold stressed at +5°C and identified 924 differentially expressed genes. Zhang et al. [27] performed comparative microarray analysis of a chilling tolerant rice cultivar (LTH; *japonica*) and a chilling sensitive rice cultivar (IR29; *indica*) and showed that although the early response to low temperatures was similar in the two cultivars, the genes that were expressed at the later time points belonged to substantially different functional categories. These studies clearly show that chilling tolerance varies among rice cultivars and that many rice genes respond to low temperature stress.

In order to gain new insights into cold stress response in rice, the main aim of this study was to conduct a global cold (+4°C) responsive gene expression profiling of the Nepalese highland rice cultivar Jumli Marshi (JM) (spp. *japonica*). This rice is grown in the district Jumla located at an altitude of up to 3,050 m in Nepal. The average maximum and minimum temperature in the region is +21°C and +4°C, respectively [28]. JM has been the preferred variety grown in this district for several decades, thus making Jumla the highest and coldest region in the world for commercial cultivation of rice. The fact that JM can be grown at this altitude while maintaining productivity corresponding to 60% of the average Nepal rice productivity per hectare [28] implies that JM is indeed chilling tolerant.

Obviously, JM has developed ways to protect itself from cold stress. Identifying genes involved in the underlying molecular mechanisms could reveal new insights into how cold tolerance is achieved. This will ultimately enable development of new cultivars with improved cold tolerance. In this work, we performed transcriptome analysis of JM under cold stress and identified 4,636 differentially expressed genes. To our understanding, this is the first report on the global analysis of cold responsive genes in Nepalese highland rice during early cold stress (<24 h, +4**°**C).

## Materials and Methods

### Plant Material and Growth Conditions

*Regular growth condition*: Seeds of two rice cultivars, *Oryza sativa,* ssp. *japonica*, cv. Jumli Marshi (JM) and ssp. *indica*, cv. IR64 (IR64), were first soaked in water for 16 hours at room temperature and thereafter grown on standard soil in 14 hours photoperiod, with a day/night air temperature of 25°C/20°C and 250 µmol m^2^ s^−1^ light.

#### Cold condition

At mid-day, three weeks old plants were transferred to growth chambers (Percival) in the same photoperiod, but with an air temperature of 4°C and a light intensity of 100 µmol m^2^ s^−1^. Pooled leaf tissue from five individual Jumli Marshi plants were harvested at 0, 0.5, 2, 4, 8, and 24 hours, frozen in liquid nitrogen and stored at −80°C until further analysis.

### Chlorophyll Fluorescence Measurements

Chlorophyll fluorescence was measured with the portable chlorophyll fluorometer PAM 2000 (Heinz Walz GmbH, Germany) and the photosystem II efficiencies F_v_/F_m_ = (F_m_–F_0_)/F_m_ were estimated as per the manufacturer’s instructions. Plants were dark acclimated for one hour before taking measurements from up to 20 individual seedlings.

### Quantitative Real-time RTPCR Measurements

TotalRNA was extracted with RNeasy plant mini kit (Qiagen, Cat. No. 74904) as per the manufacturer’s instructions. DNAse digestion was performed on-column as per the instructions using RNase-Free DNase Set (Qiagen, Cat. No. 79254). The primer sets used in the study were DREB1aF 5′GGACCTGTACTACGCGAGCTT3′, DREB1aR 5′GGG AAA ATT GTA CAG TTG ATT GA3′, DREB1bF 5′AGC TCG CCG GCT CCG ACA3′, DREB1bR 5′GGG AGA ATT CTG GCA CAT TCC3′, DREB1cF 5′GAG TTG GAG CTA GCA GTT TTG AG3′, DREB1cR 5′TAG CTG TAT AGG AGG AGC AAA GC3′, OsActin1F 5′ATC CTT GTA TGC TAG CGG TCG A3′, OsActin1R 5′ATC CAA CCG GAG GAT AGC ATG3′ [29], [30]. Quantitative real-time PCR was performed on biological triplicates with the default protocol in iScript One-Step RT-PCR kit with SYBR Green kit (BioRad, Cat. No. 170-8893) using BioRad C1000 Thermal Cycler. Relative expression of the genes was calculated with the Pfaffl method [31].

### Data Preparation and Analysis

Total RNA was extracted from JM leaf tissue with TRIZOL reagents (Invitrogen) according to the manufacturer’s protocol and purified by RNeasy MinElute Cleanup Kit (Qiagen). The RNA quality and concentration was measured using Agilent 2100 BioAnalyzer and Nanodrop ND-1000. Biotinylated target cRNA was prepared from 4 µg of total RNA following the manufacturer’s specifications (Affymetrix). The samples were then hybridized to Affymetrix GeneChip® Rice Genome Arrays, which contain probes to query ∼51K transcripts representing both *japonica* and *indica* cultivars. The chips were thereafter washed and stained in a GeneChip® Fluidics Station 450. Scanning was carried out with GeneChip® Scanner 3000 and image analysis was performed using GeneChip® Operating Software. Two biological replicates were analysed per time point. The CEL files were submitted to ArrayExpress with the accession number E-MEXP-3718.

Data was processed using Bioconductor [32] in R v2.14. Raw CEL files were background corrected with the GCRMA method and quantile normalized using the Bioconductor package affyPLM v1.30 [33]. Probe-set present/absent calls were calculated with the mas5calls method in the AffyBatch package [34]. Probe sets that were marked present in at least one of the samples were considered for further analysis. Probe sets with IQR greater than the median IQR of all probe sets were selected with the genefilter v1.36 package. To identify differentially expressed probe sets, a linear contrast matrix was built between the control and the cold-treated samples using the Limma v3.10 package [35]. Differentially expressed genes (DEGs) were identified with the empirical Bayes method in the Limma package using default parameter settings. A Benjamini-Hochberg corrected p-value <0.05 was set as the significance threshold. Thereafter, only probe sets with a unique RAP OS ID were retained for further analysis.

MapMan annotations for Oryza sativa (spp. *japonica*) genes (v1.0) were downloaded (www.mapman.gabipd.org), and annotations for the DEGs were extracted using a custom Perl script. Hierarchical clustering was performed with Cluster 3.0, using the average linkage method and Pearson correlation as similarity measure. Dendrograms were generated with Java TreeView 1.1.6. Clustering was also performed with Short Time-series Expression Miner (STEM, version 1.3.8) [36] using default parameters.

## Results

### JM has Higher Cold Tolerance than IR64

**Figure 1:**
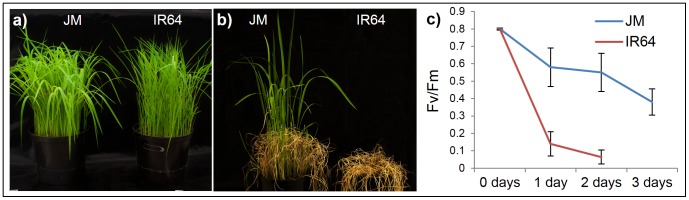
Cold stress survival in Jumli Marshi (JM) and IR64. Jumli Marshi (JM) and IR64 seedlings were grown for three weeks under regular growth conditions (see Materials and Methods) and then moved to +4°C (cold conditions). After three days in cold conditions, plants were moved back to regular growth conditions and allowed to recover for two weeks. (a) Plants just before cold exposure; (b) Cold treated plants after recovery for two weeks; JM, left; IR64, right; (c) Chlorophyll fluorescence in JM and IR64 undergoing cold stress.

To confirm the higher cold tolerance in JM compared to lowland rice, three weeks old seedlings grown at regular conditions were transferred to cold conditions and exposed to +4°C for three days (see Materials and Methods). Plants were then allowed to recover for two weeks in regular growth conditions, and their viability was visually estimated. The results confirmed that JM is chilling tolerant while IR64 is not since all IR64 plants wilted and died while the JM plants recovered from the cold stress (Figure 1a, 1b).

To quantify the stress levels in the two cultivars, chlorophyll fluorescence signals were measured. The ratio between the variable (*F*_v_) and maximum (*F*_m_) fluorescence signals reflects the efficiency of photosystem II. Plants were first dark-acclimated for 1 hour, and the fluorescence was measured (PAM 2000 fluorometer; Heinz Walz GmbH, Germany) just before transferring the plants to +4°C (0 days), and then after 1, 2 and 3 days in the cold. Readings from 50 individual seedlings were collected, and the experiment was repeated twice. Prior to cold exposure, *F*_v_/*F*_m_ ratios were similar (∼0.8) in both JM and IR64 plants, indicating that all plants were physiologically healthy. After one day in cold, the *F*_v_/*F*_m_ ratio in IR64 fell to 0.14, while, in JM, the decrease was relatively small (0.64). The differences between the averages were significant at *p*<0.0001 (Student’s t-test). After two days in cold, the *F*_v_/*F*_m_ ratio reached near zero in IR64, while, in JM, it reached 0.55 (Figure 1c). These results show that JM tolerates cold stress significantly better than IR64.

### Identification of Differentially Expressed Genes under Cold Stress in JM

Since JM is chilling tolerant, it was chosen for global gene expression profiling under cold stress. Three weeks old plants grown under regular conditions were moved to cold conditions (+4°C) at mid-day and pooled leaf tissues from five individual plants were harvested at six different time points: 0, 0.5, 2, 4, 8 and 24 hours.

**Figure 2:**
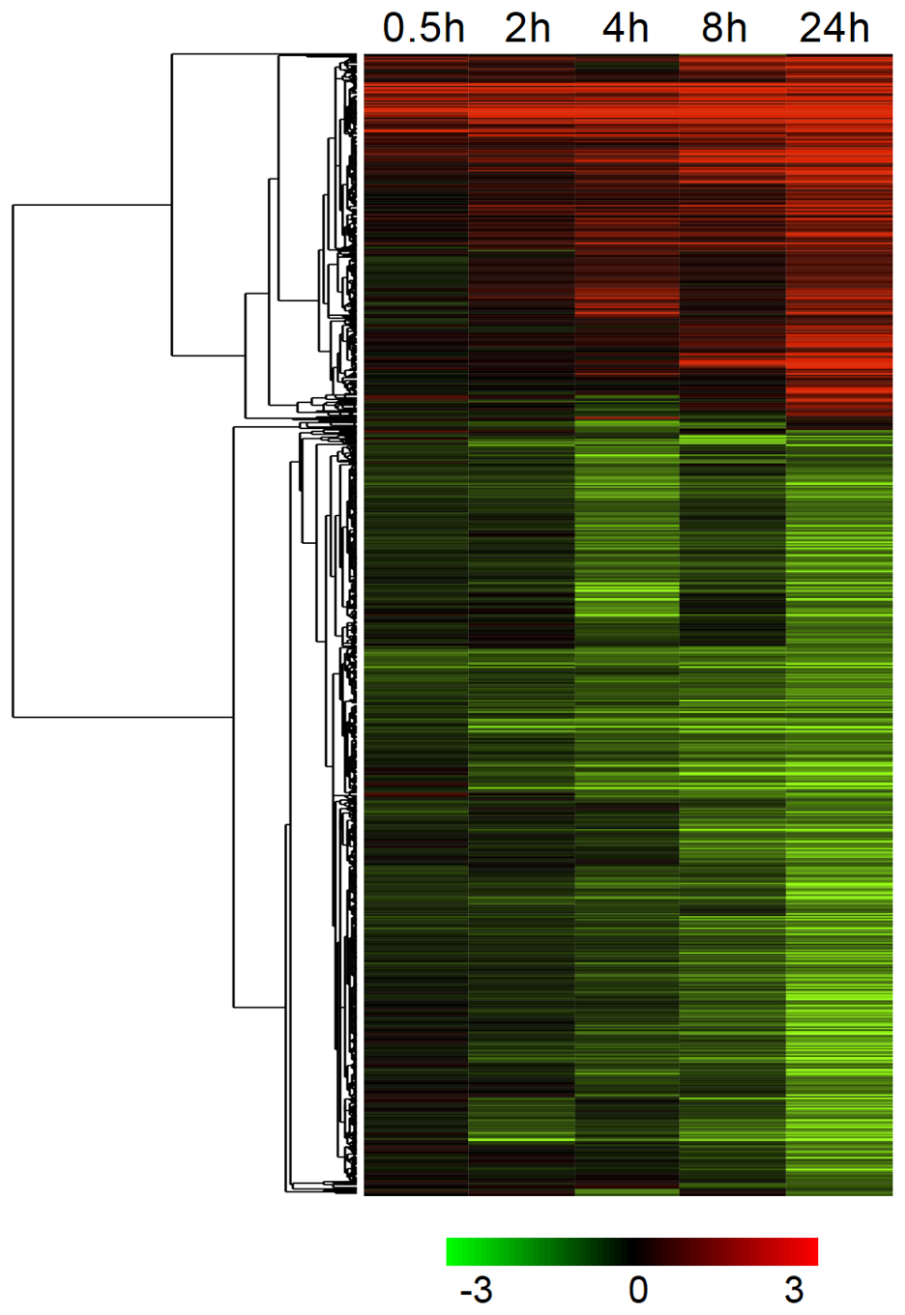
Hierarchical clustering of JM genes significantly differentially expressed in at least one time point under cold stress. Clustering was performed with Cluster 3.0, using the average linkage method and Pearson correlation as similarity measure. The tree was generated with Java TreeView 1.1.6. Red: up-regulated genes; green: down-regulated genes. Log_2_ transformed fold change expression levels are indicated by the color gradient. The extreme values in the color gradient are −3 to +3 in log_2_ scale.

**Figure 3:**
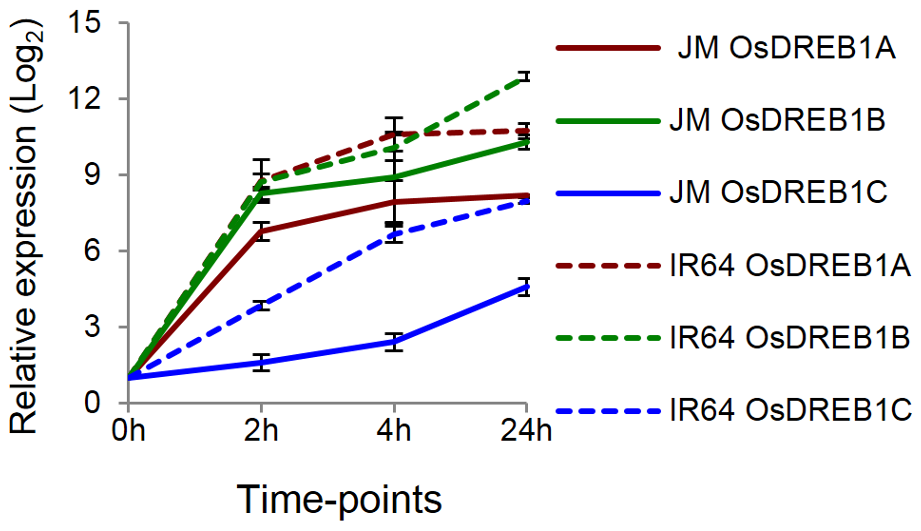
Monitoring Os*DREB1* mRNA levels in JM and IR64 by quantitative real-time RT-PCR. Relative expression levels of Os*DREB1a*, OS*DREB1b* and Os*DREB1c* at 0 h, 2 h, 4 h and 24 h measured by quantitative real-time RT-PCR measured in biological triplicates. Solid line represents JM and dashed line represents IR64.

Microarray data analysis revealed that 4,636 genes were significantly differentially expressed (Table S1). The complex transcriptional response pattern to early cold stress in JM can be visualized by hierarchical clustering (Figure 2). Two major clusters were formed, one with 1,490 up-regulated genes, and the other with 3,146 down-regulated genes. There were 183 genes (148 up, 35 down) DE by at least two folds (log_2_) within two hours of cold exposure. Most common annotations in this set were genes with unknown function (83 genes), regulation of transcription (36 genes), protein degradation or post-translational modification (16 genes) and signaling (8 genes) (Figure S1). Out of the 36 genes with transcription factor (TF) activity, the most common TF families were AP2/EREBP (12 genes), WRKY (5 genes) and bHLH (4 genes). The most highly induced genes within 2 hours were *OsDREB1B* (10 log_2_ folds) and *OsDREB1A* (8.9 log_2_ folds). Quantitative real-time RT-PCR measurements of six genes (Figure 3 and Figure S2) showed good correlation with the microarray data.

### *DREB1* Genes are Similarly Expressed in JM and IR64

*DREB1*s encode transcription factors that belong to the AP2 family of DNA binding proteins. Several experiments in many different plant species have shown that *DREB1A*, *DREB1B* and *DREB1C* are induced within 30 minutes of exposure of plants to cold. For example, over-expression of *DREB1*s in *Arabidopsis* resulted in increased freezing tolerance of non-acclimated transgenic plants compared to the non-transformed control plants [37]. Thus, the mRNA expression levels of the three *DREB1* genes were measured by quantitative RT-PCR in JM and IR64. The results revealed that all three genes were highly induced (>5 folds) within 2 hours in both JM and IR64, although, the expression levels were higher in IR64 than in JM (Figure 3). Previously Zhang et al [38] identified 22 genes as part of the OsDREB1c regulon. In JM, 18 of these 22 genes were DE upon cold stress indicating the critical role of OsDREB1c regulon under cold tolerance in JM (Figure S3).

### Comparative Analysis of Microarray Data from Four Rice Cultivars Reveals Genes that are Differentially Expressed only in Chilling Tolerant Rice

Comparative analysis with previously published rice microarray data was done to identify genes that are differentially expressed (DE) only in chilling tolerant rice. Zhang *et al.* [27] performed microarray data analyses of the chilling tolerant rice cultivar LTH (spp. *japonica*) and the chilling sensitive rice cultivar IR29 (spp. *indica*). Mittal *et al.* [26] performed microarray data analysis of the rice cultivar Pusa Basmati (PB1, ssp. *indica*). PB1 is mostly cultivated in the North-Western part of India [39] in the warm season May-November and thus is considered chilling sensitive.

**Figure 4:**
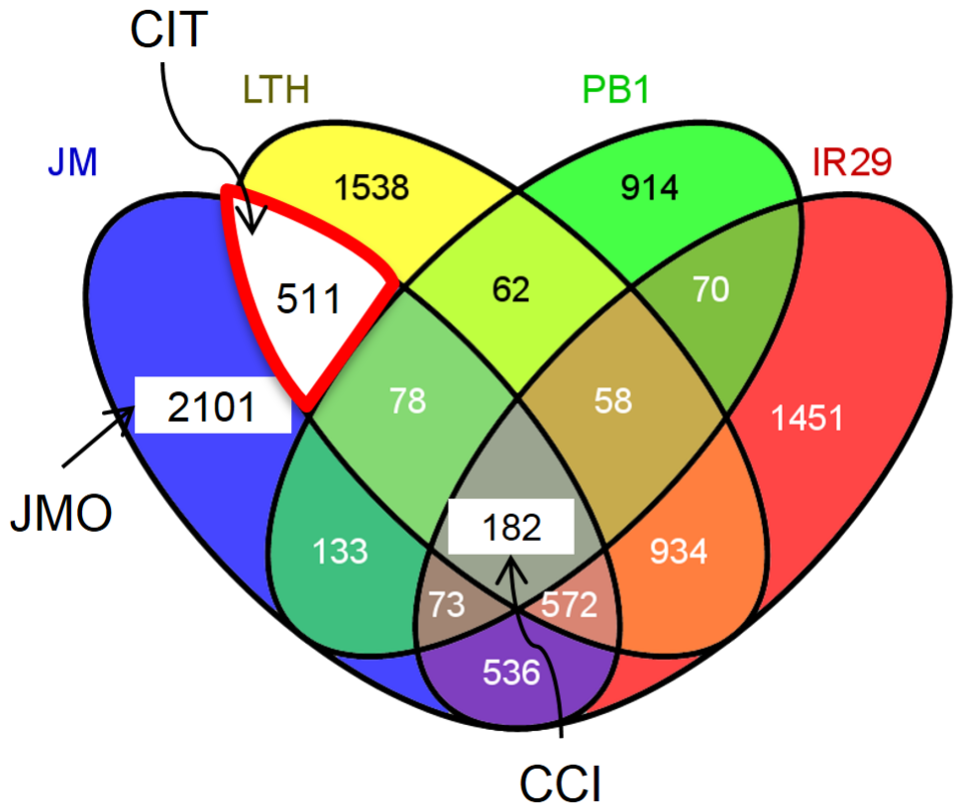
Number of DE genes in four rice cultivars. Comparison of DE genes in four rice cultivars upon cold stress. CCI (Common Cold Induced), CIT (Cold Induced in Tolerant cultivars), JMO (Jumli Marshi Only). Venn diagram created using VENNY [71].

In this work, comparative analysis was done between JM and the three previously published datasets and the genes that were DE by at least 2 folds in at least one of the four rice cultivars were studied further (Figure 4). The analysis identified 182 genes that were DE by at least two folds in all four rice cultivars. These genes were termed Common Cold Induced (CCI) (Figure 4, Table 1). The CCI gene list includes several well-known cold induced genes induced by 2 folds within 2 hours after induction including OsDREB1C, OsDREB1G, OsWRKY71, OsNAC3 and also genes induced later than 2 hours including OsWRKY1 and OsNAC4. The comparative analysis also identified 511 genes that were only expressed in the two chilling tolerant cultivars JM and LTH. These 511 genes were termed as Cold Induced in Tolerant cultivars (CIT). Expression levels of three genes from the CIT group were validated by quantitative RT-PCR (Figure S2). As the CIT genes are DE only in the chilling tolerant cultivars, they may play a significant role in chilling tolerance in rice. A third set with 2,102 genes was only DE in JM. These were denoted as JM Only (JMO). As JM is cultivated at altitudes of up to 3,050 m, further understanding of the JMO genes may help to identify novel mechanisms for survival of plants in these environmental conditions. Therefore, the JMO genes were further analyzed in this study. A complete list of genes in different groups is in Table S2.

**Table 1:** CCI (Common Cold Induced) genes differentially expressed in all four rice cultivars.

Os_RAPDB_ID	0.5 h	2 h	4 h	8 h	24 h	Symbol	Bin	MapMan description
Os02g0677300	5.4	5.7	6.3	7.6	7.6	OsDREB1G	27.3.3	RNA.regulation of transcription.AP2/EREBP
Os01g0186900	5.1	6.9	7.3	7.9	9.0		35.2	not assigned.unknown
Os10g0391400	4.9	4.7	5.7	7.0	6.5		35.2	not assigned.unknown
Os08g0482600	4.8	3.9	2.9	6.6	5.4		26.19	misc.plastocyanin-like
Os12g0150200	4.0	3.7	5.1	7.3	7.2		26.1	misc.cytochrome P450
Os10g0562900	3.9	5.1	5.6	6.0	6.6		27.3.3	RNA.regulation of transcription.AP2/EREBP
Os01g0955100	3.9	4.6	4.5	5.2	6.2		30.3	signalling.calcium
Os02g0629000	3.8	4.0	3.9	5.4	7.5		35.2	not assigned.unknown
Os02g0134200	3.6	5.4	6.2	6.9	8.4		35.2	not assigned.unknown
Os02g0703600	3.4	5.5	6.3	7.4	8.5	OsABA8OX1	26.1	misc.cytochrome P450
Os02g0181300	3.2	3.5	4.0	5.2	5.8	OsWRKY71	27.3.32	RNA.regulation of transcription.WRKY
Os04g0301500	3.2	2.1	2.9	5.4	5.4		27.3.6	RNA.regulation of transcription.bHLH,Basic Helix-Loop-Helix family
Os03g0741100	3.1	2.0	2.4	4.7	4.6	OsbHLH148	27.3.6	RNA.regulation of transcription.bHLH,Basic Helix-Loop-Helix family
Os01g0826400	3.1	2.1	2.2	4.2	5.1	OsWRKY24	27.3.32	RNA.regulation of transcription.WRKY
Os01g0862800	3.0	1.7	1.0	4.7	5.0		27.3.27	RNA.regulation of transcription.NAC
Os03g0181100	2.9	3.9	4.5	5.4	5.4	OsJAZ4	35.2	not assigned.unknown
Os07g0680600	2.9	3.5	3.2	4.1	4.3		35.1	not assigned.no ontology
Os03g0191900	2.9	2.5	2.2	2.9	3.3		27.3.3	RNA.regulation of transcription.AP2/EREBP
Os03g0815100	2.8	3.6	4.9	6.0	6.7		27.3.27	RNA.regulation of transcription.NAC
Os07g0225300	2.8	3.9	4.7	5.7	6.2	OsNAC3	27.3.27	RNA.regulation of transcription.NAC
Os06g0133500	2.8	2.3	2.6	3.3	2.8		35.2	not assigned.unknown
Os02g0759400	2.7	2.6	3.4	5.0	5.7		29.5.11.4.2	protein.degradation.ubiquitin.E3.RING
Os04g0583200	2.6	3.5	3.5	4.5	6.0		35.2	not assigned.unknown
Os03g0183500	2.6	1.6	3.1	4.7	4.4		33.99	development.unspecified
Os04g0517100	2.5	3.1	4.5	5.7	5.9		27.3.25	RNA.regulation of transcription.MYB
Os01g0863300	2.5	0.9	0.7	3.8	4.5		27.3.26	RNA.regulation of transcription.MYB
Os02g0756800	2.5	5.2	6.1	6.8	7.2		30.1	signalling.in sugar and nutrient physiology
Os03g0315400	2.5	4.3	4.6	6.1	5.7		27.3.25	RNA.regulation of transcription.MYB
Os03g0820400	2.5	4.2	5.8	6.8	8.3		27.3.11	RNA.regulation of transcription.C2H2
Os03g0107700	2.4	3.7	4.8	5.5	6.3	Orysa; EL2	35.2	not assigned.unknown
Os02g0758200	2.4	2.8	3.5	3.9	5.4		35.2	not assigned.unknown
Os10g0392400	2.4	1.9	2.4	4.1	4.9	OsJAZ1	35.2	not assigned.unknown
Os01g0816100	2.4	1.8	2.4	4.1	5.1	OsNAC4	27.3.27	RNA.regulation of transcription.NAC
Os01g0864500	2.3	2.7	3.0	4.0	3.8		35.1	not assigned.no ontology
Os01g0389700	2.2	2.5	0.2	3.8	3.9		35.2	not assigned.unknown
Os01g0135700	2.2	3.2	3.4	4.2	4.8		30.3	signalling.calcium
Os04g0543900	2.1	5.1	6.2	7.4	7.5	OsGDH2	12.3.1	N-metabolism.N-degradation.glutamate dehydrogenase
Os09g0385700	2.1	2.1	1.5	2.2	2.4		27.3.99	RNA.regulation of transcription.unclassified
Os06g0127100	2.1	4.1	5.4	7.2	6.8	OsDREB1C	27.3.3	RNA.regulation of transcription.AP2/EREBP
Os09g0325700	2.0	0.1	−0.1	−0.2	0.0		29.4	protein.postranslational modification
Os01g0727500	2.0	2.4	3.8	3.9	5.0		35.2	not assigned.unknown
Os01g0855600	2.0	4.5	5.1	5.9	6.4		35.2	not assigned.unknown
Os03g0152000	1.9	2.7	3.9	4.5	6.4		15.2	metal handling.binding, chelation and storage
Os03g0152000	1.9	2.7	3.9	4.5	6.4		34.99	transport.misc
Os08g0474000	1.9	2.5	4.0	5.9	6.4		27.3.3	RNA.regulation of transcription.AP2/EREBP
Os01g0246700	1.9	0.8	1.1	3.0	4.0	OsWRKY1	27.3.32	RNA.regulation of transcription.WRKY
Os05g0545400	1.9	4.6	4.4	6.5	7.8		29.4	protein.postranslational modification
Os03g0188500	1.9	2.8	3.8	5.2	6.8		35.2	not assigned.unknown
Os05g0380900	1.8	3.6	3.8	4.1	4.9		30.3	signalling.calcium
Os01g0905200	1.8	2.5	3.2	4.5	6.1		31.4	cell.vesicle transport
Os04g0497000	1.8	2.9	3.1	4.7	5.4		26.7	misc.oxidases - copper, flavone etc.
Os03g0180800	1.8	3.1	4.5	5.1	5.4	OsJAZ3	35.2	not assigned.unknown
Os06g0662200	1.7	0.7	1.3	1.0	3.1		27.3.35	RNA.regulation of transcription.bZIP transcription factor family
Os04g0372700	1.7	3.5	3.8	4.7	5.9		26.7	misc.oxidases - copper, flavone etc.

_2_) at 0.5 hr. Bin: MapMan bin ID. Genes are sorted on their expression values (log

#### Identification of over-represented cis-elements in CCI and CIT gene sets

**Figure 5:**
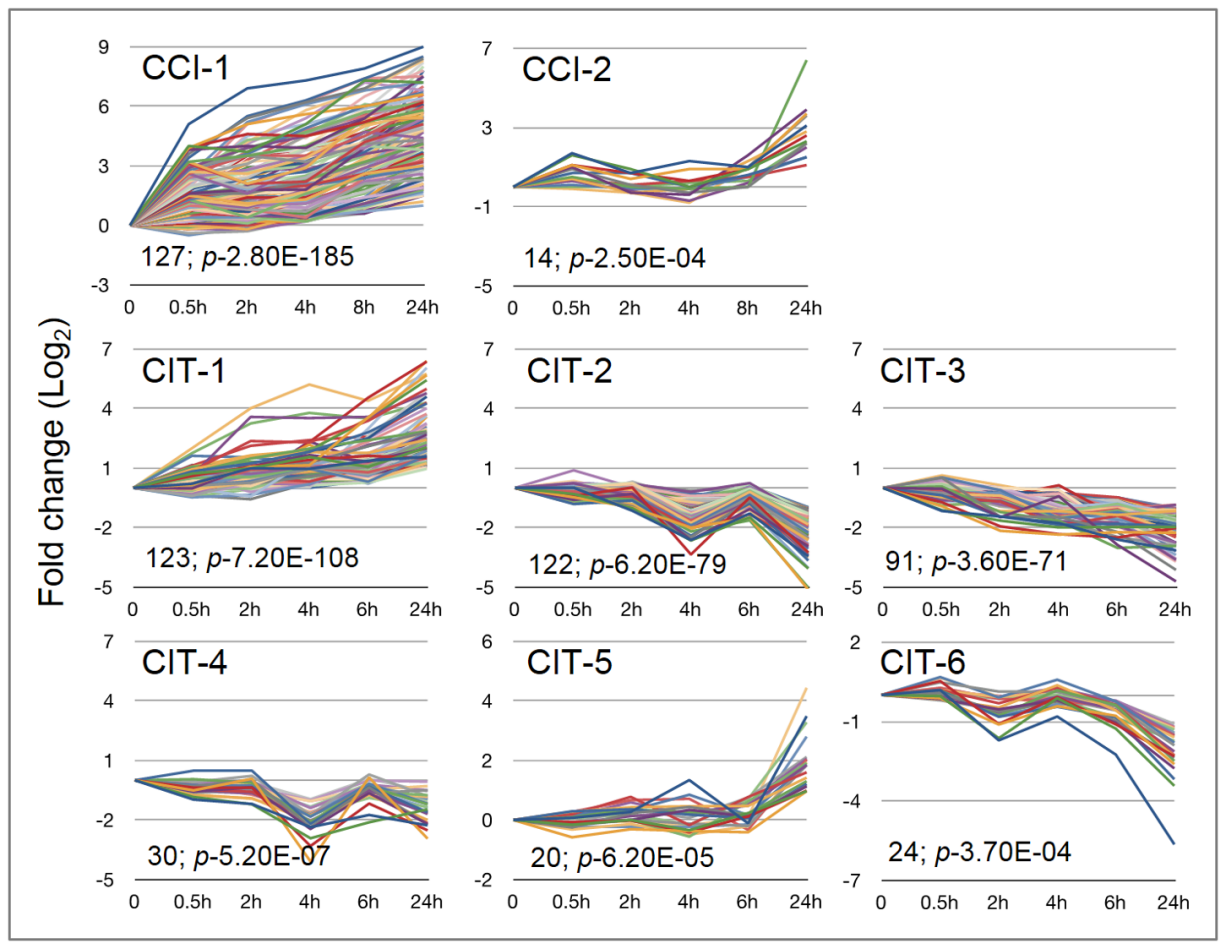
STEM clustering of CCI and CIT gene sets. STEM clustering identified two significant clusters in the CCI gene set and six significant clusters in the CIT gene set.

Clustering was performed with STEM [36] to identify the CCI and CIT genes that were significantly co-expressed. The clustering of CCI genes resulted in two statistically significant clusters with 127 and 14 genes each, whereas the CIT genes were grouped into six clusters (Figure 5). Promoter analyses of genes in the eight clusters by the Osiris tool [40] lead to the identification of over-represented cis-elements/motifs previously known to be involved in cold regulation (Table 2). For example, CCI cluster 1 contains the well-known cold regulated motifs ABRE and DRE/CRT, and eleven other previously known motifs. This suggests a cross-talk between various transcription factor families during cold stress. Among the six CIT clusters, known over-represented motifs were only identified in cluster 3. In the remaining five clusters, no previously known motifs could be identified, suggesting that yet unknown regulons are involved in regulating many of the CIT genes.

**Table 2:** Previously known significantly over-represented (*p*<0.01) cis-elements/motifs in CCI (Common Cold Induced) and CIT (Cold Induced in Tolerant cultivars) clusters.

Cis-element	Consensus sequence	#Pro	*p*	Description
**CCI Cluster 1**				
ABREOSRAB21	ACGTSSSC	52	10^−5^	ABA responsive element (ABRE) of wheat Em and rice (O.s.) rab21 genes
motifB	TACGTGTC	14	10^−4^	Similar to ABRE element
DRECRTCOREAT	RCCGAC	83	10^−3^	Core motif of DRE/CRT cis-acting element
ACGTABREMOTIFA2OSEM	ACGTGKC	58	10^−3^	ACGT-core of motif A in ABRE of the rice gene
CGACGOSAMY3	CGACG	105	10^−3^	CGACG element found in the GC-rich regions of the rice
G-box-like	CACGTG	56	10^−3^	Found in rice sucrose phosphate synthase gene promoter
ACGTOSGLUB1	GTACGTG	34	10^−3^	Required for endosperm-specific expression
GCrichrepeatII	CGCCGCGC	30	10^−3^	Found in RAB21 promoter
MYB1AT	WAACCA	117	10^−3^	Found in promoter of chymotrypsin inhibitor-like 1 gene
ABREmotif	TGACGT	63	10^−3^	Found in promoter of 6-phosphogluconate Dehydrogenase;
SITEIOSPCNA	CCAGGTGG	12	10^−3^	Site I of rice (O.s.) PCNA (proliferating cell nuclear antigen) gene
**CCI Cluster 2**				
E2F1OSPCNA	GCGGGAAA	4	10^−3^	Involved in transcriptional activation in actively dividing cells and tissue
ACGTABREMOTIFA2OSEM	ACGTGKC	10	10^−3^	
**CIT Cluster 3**				
ABRE3OSRAB16	GTACGTGGCGC	2	10^−3^	ABA-responsive element of rice
MYCATERD1	CATGTG	69	10^−2^	Found in promoter of chymotrypsin inhibitor-like 1 gene
MYCATRD22	CACATG	69	10^−2^	Found in promoter of chymotrypsin inhibitor-like 1 gene
WUSATAg	TTAATGG	28	10^−3^	Target sequence of WUS in the intron of AGAMOUS gene in Arabidopsis

[40]. #Pro: Number of promoters with the motif. Over-represented motifs were identified in 2 kb upstream of start codon using Osiris

To identify new putative motifs, gene promoters from CIT clusters 1, 2, 4, 5 and 6 were analyzed using the Element algorithm [41]. The 1 kb upstream region was analyzed for each promoter, and the top five hits were retained (Table 3). This analysis showed that while a sub-set of the over-represented words were entirely or partially similar to previously known cis-elements, the remaining words did not belong to any previously known cis-elements and thus are newly identified putative cis-elements (Table S3).

**Table 3:** Over-represented words in the 1 kb upstream promoter regions were identified using the Element algorithm [41].

Group	Words	Known cis-elements	
**CIT Cluster 1**			
1	6	HEXAT, SORLIP1AT, ABRELATEERD1, ACGTATERD1, BOXIIPCCHS	
2	22	ABRELATERD1, ACGTATERD1, CACGTGMOTIF, IRO2OS, T/GBOXATPIN2	
3	26	POLLEN1LELAT52, GT1GMSCAM4, DOFCOREZM, TATABOX5	
4	13	CURECORECR	
5	9	None	
**CIT Cluster 2**			
1	25	GT1GMSCAM4, DOFCOREZM	
2	16	WBOXATNPR1, WRKY71OS	
3	12	None	
4	9	None	
5	13	IBOX	
**CIT Cluster 4**			
1	2	None	
2	6	SORLIP2AT, ABFOS, HY5AT	
3	6	None	
4	4	None	
**CIT Cluster 5**			
1	2	None	
2	9	None	
3	4	None	
4	4	None	
5	1	None	
**CIT Cluster 6**			
1	18	None	
2	18	ACGTATERD1	
3	7	DOFCOREZM, TAAAGSTKST1, ACGTATERD1	
4	11	GT1GMSCAM4	
5	8	None	

Group: Group of significantly over-represented words in the promoter regions; Words: No. of significant words in each group, words can be of different lengths; Known cis-elements: Words found in the previously known motifs.

#### Comparative analysis of functional annotations

**Figure 6:**
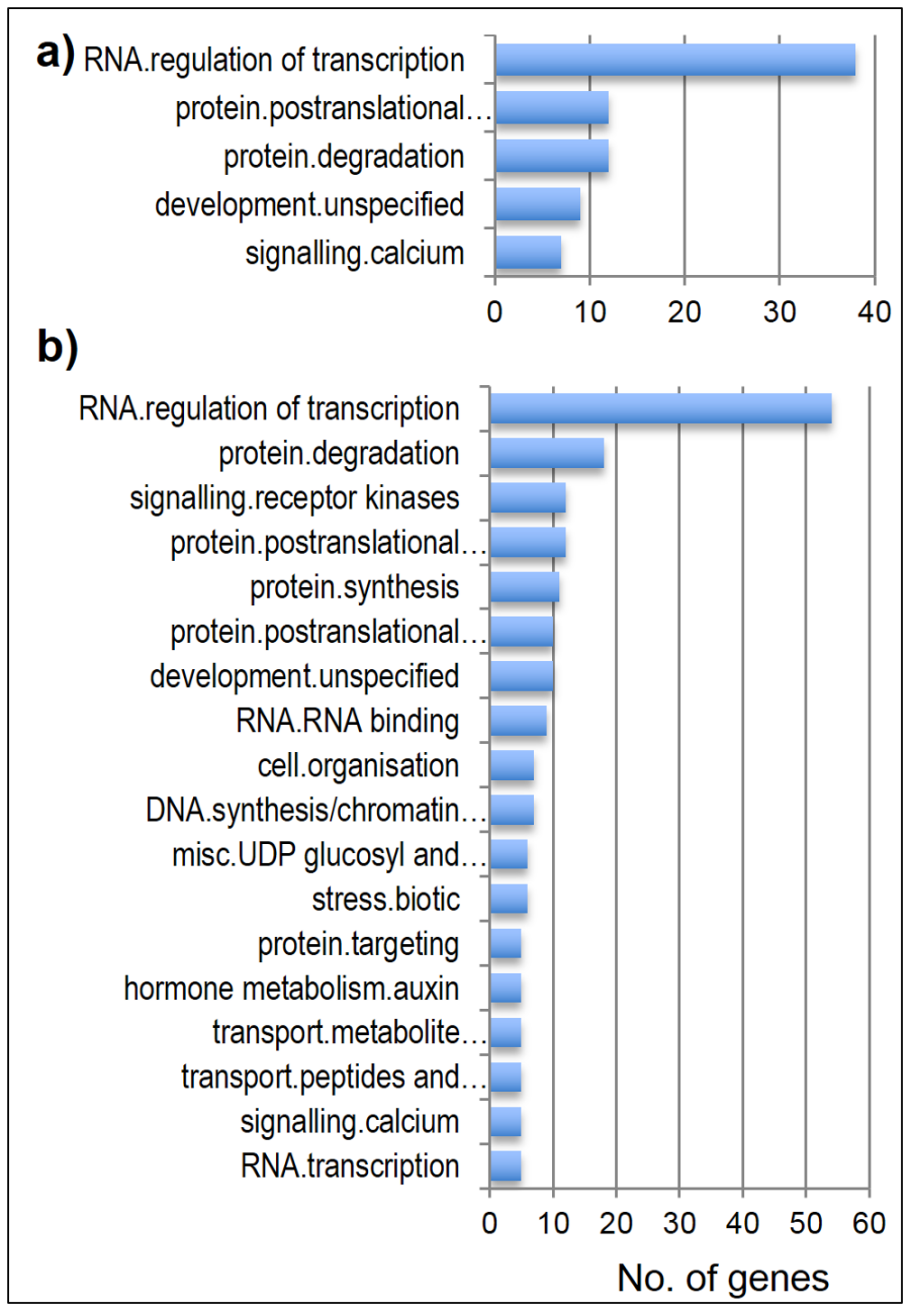
Functional annotations in CCI and CIT gene sets. MapMan annotations in CCI and CIT gene sets. Categories with at least two genes are shown. a) CCI gene set; b) CIT gene set.

**Figure 7:**
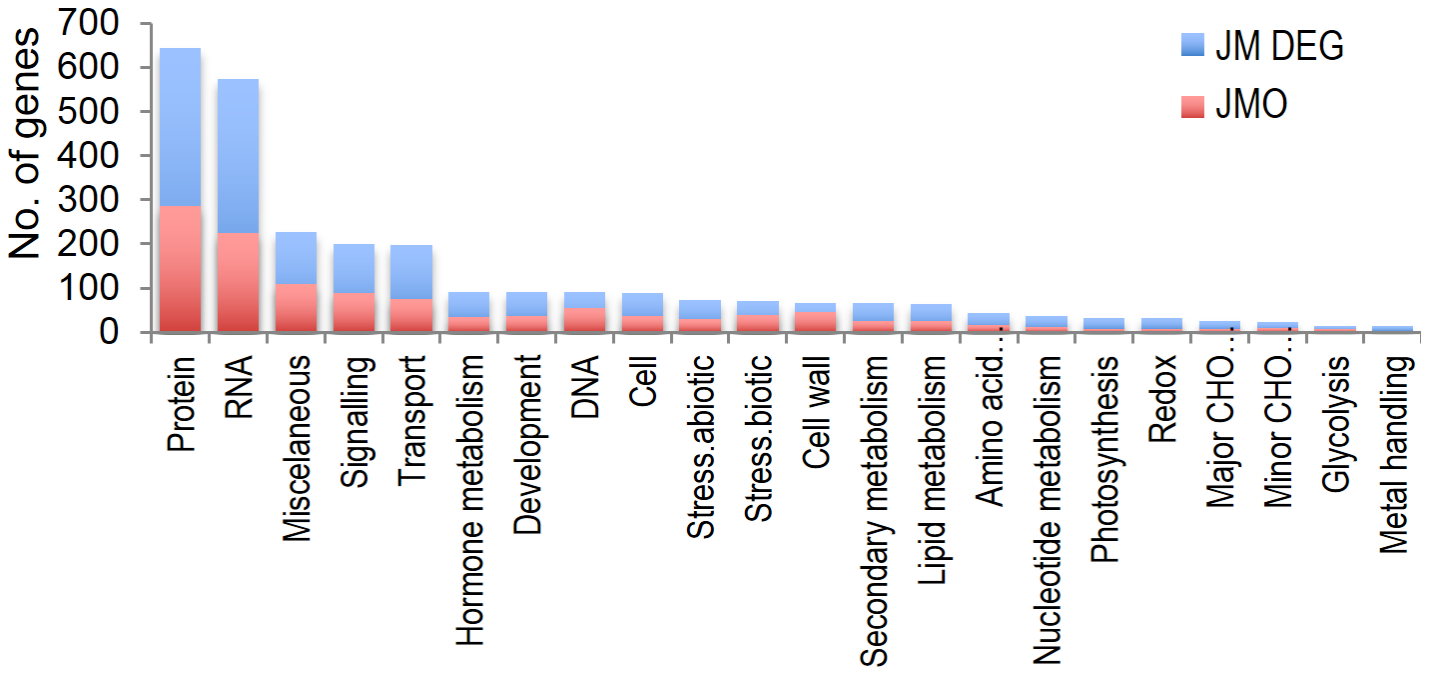
Functional annotations of DE genes in JM. Only the top-level category of the MapMan annotation for each gene was considered for simplicity. Blue: all DEGs in JM; Red: JMO. Categories with at least 10 genes are shown. Genes with unknown annotations are not included in the graph.

**Figure 8:**
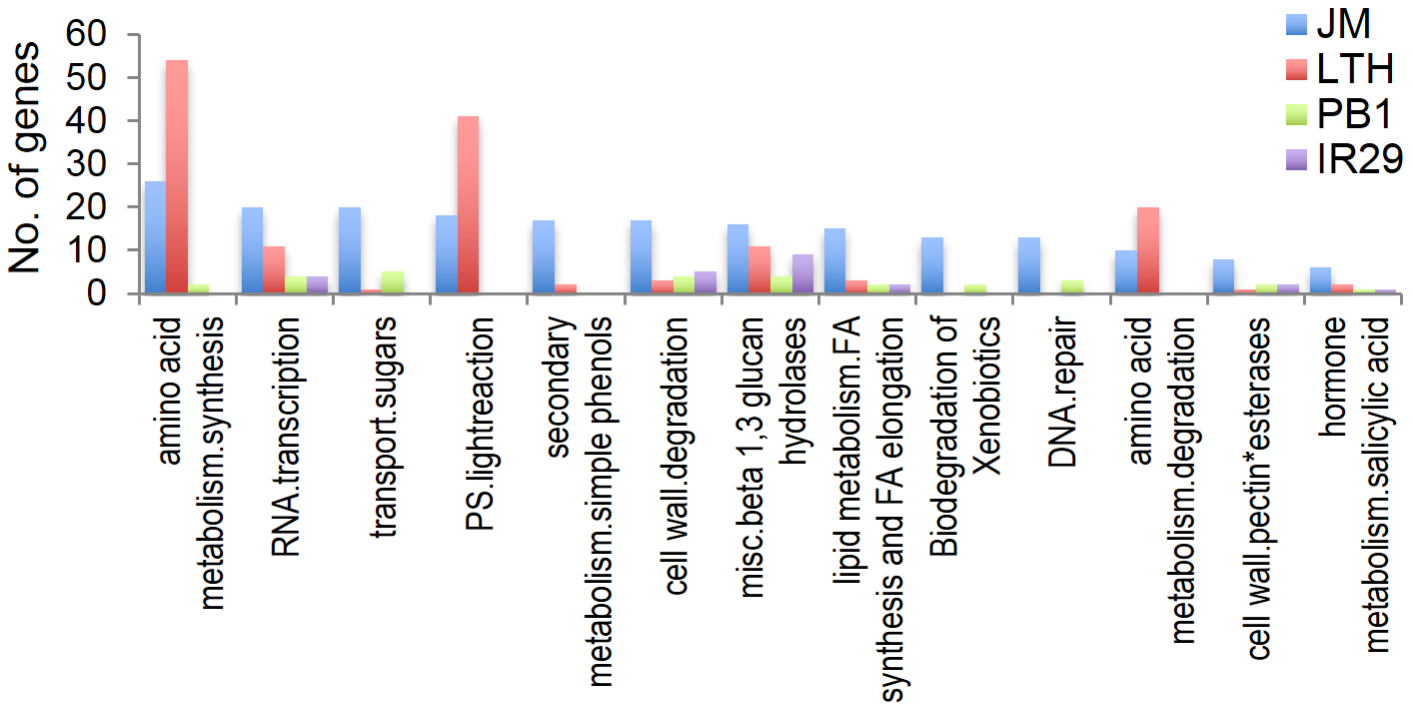
Highly abundant annotations in JM. MapMan annotations that were at least five times more abundant in JM compared to PB1 and IR29.

MapMan annotations common in the CCI and CIT gene sets were identified. Examples of such annotations are regulation of transcription, post-translational modification, protein degradation, development and calcium signaling. Interestingly, the CIT gene set also included additional annotations such as signaling in receptor kinases, protein synthesis and RNA binding (Figure 6). MapMan annotations were also obtained for all 4,636 DEGs (Table S4). The top three annotations were protein (645), RNA (574) and miscellaneous (227) (Figure 7). Analysis of annotations that were highly abundant in JM and LTH compared to chilling sensitive PB1 and IR29 showed that amino acid metabolism, RNA transcription, transport of sugars, photosynthesis light reaction and secondary metabolisms were the top five over-represented annotations in the cold tolerant cultivars (Figure 8).

### Several TF Families are DE in JM

**Figure 9:**
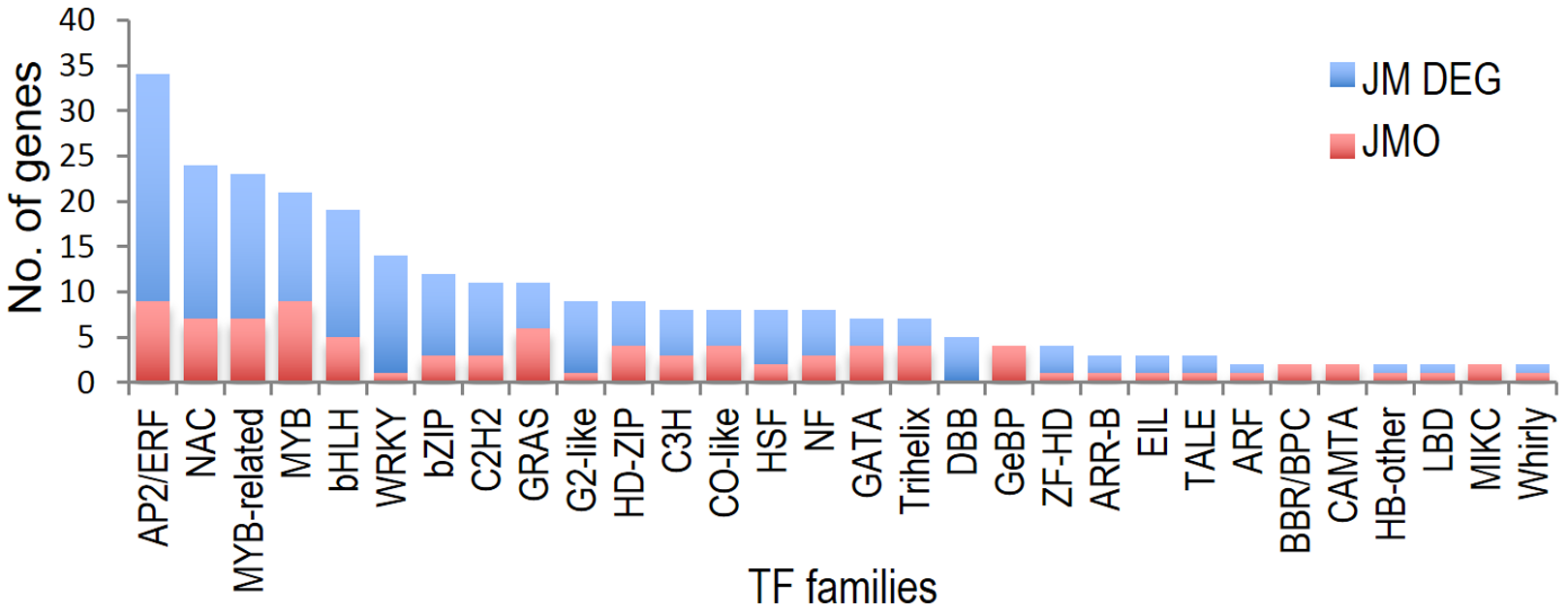
Differentially expressed transcription factor (TF) encoding genes. TF encoding genes were identified from the Plant Transcription Factor database (PlantTFDB).

**Figure 10:**
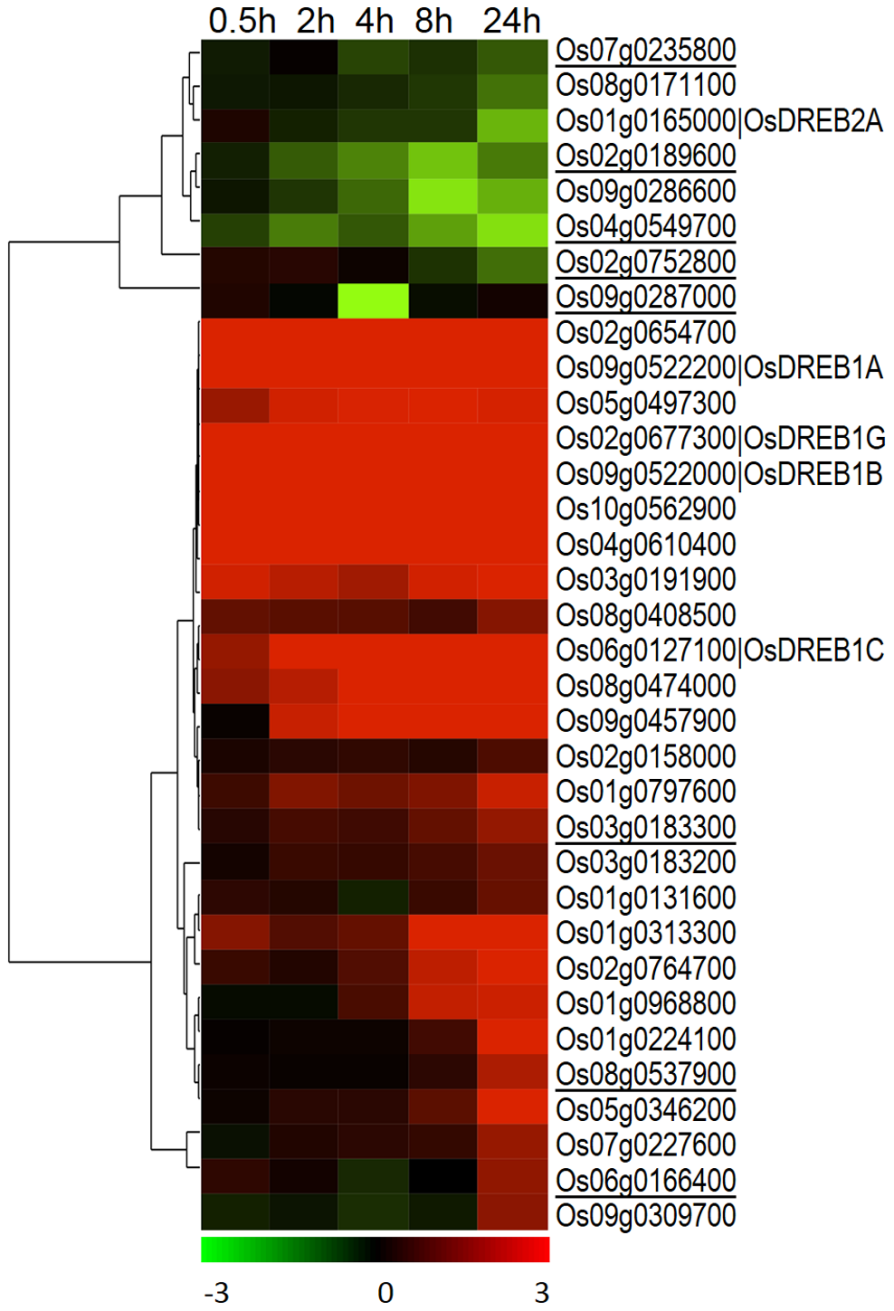
Clustering of AP2/ERF domain containing genes. Hierarchical clustering of differentially expressed AP2 domain containing genes. JMOs are underlined.

Annotations were also downloaded from the Plant Transcription Factor database (PlantTFDB) [42] to assess the most prominent cold responsive transcription factor (TF) families. In JM, 277 (5.9%) of the DEGs belonged to 37 represented TF families. TFs from well-known cold stress associated families like AP2/ERF (34 genes), NAC (24 genes), MYB-like (21 genes), bHLH (19 genes) and WRKY (14 genes) were among the over-represented families (Figure 9, Table S1). Hierarchical clustering of the AP2/ERF TFs shows that most of these genes are either highly up- or down-regulated within 24 hours of cold stress and thus form two major clusters based on their expression (Figure 10). *OsDREB1*s are among the most highly induced genes within 30 minutes of exposure to cold.

### Significant Changes Occur in Hormone, Signaling and Carbohydrate Metabolism Associated Genes

**Figure 11:**
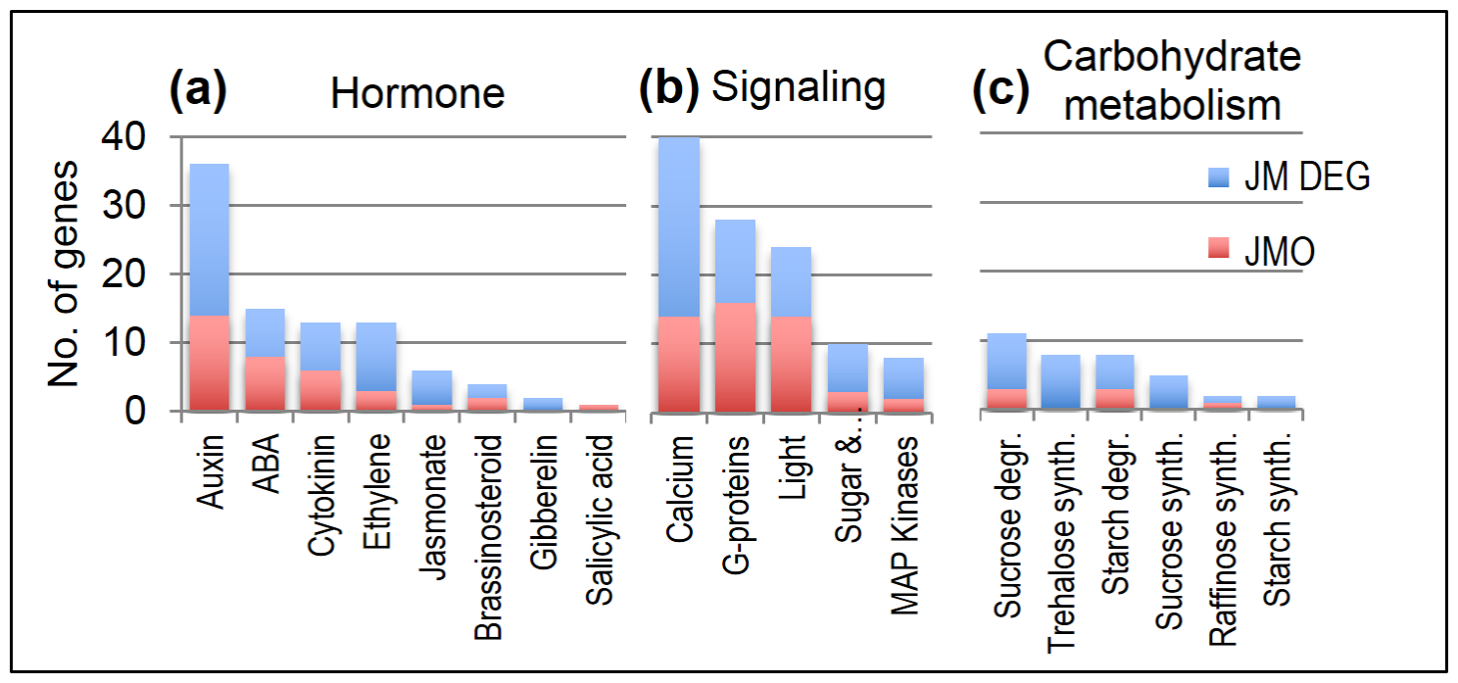
Genes in hormone, signaling and carbohydrate metabolism pathways. Number of cold induced genes involved in various pathways, as identified from MapMan annotations.

A number of signaling, as well as hormone regulation pathways, have previously been shown to be prominent in cold stress responses [43], [44]. Genes involved in different hormone pathways were identified from the MapMan annotations. Among pathways coupled to cold stress were auxin (indole-3-acetic acid) (36 genes), abscisic acid (ABA) (15 genes), cytokinin (13 genes) and ethylene (13 genes) (Figure 11a). Genes related to calcium signaling (40 genes) and the MAP kinase cascade (8 genes) were also differentially expressed (Figure 11b), as well as genes involved in sugar synthesis and degradation pathways, including sucrose degradation (11 genes), trehalose synthesis (8 genes) and starch degradation (8 genes) (Figure 11c).

### Cold Stress Activates ROS Scavenging Mechanisms in JM

**Figure 12:**
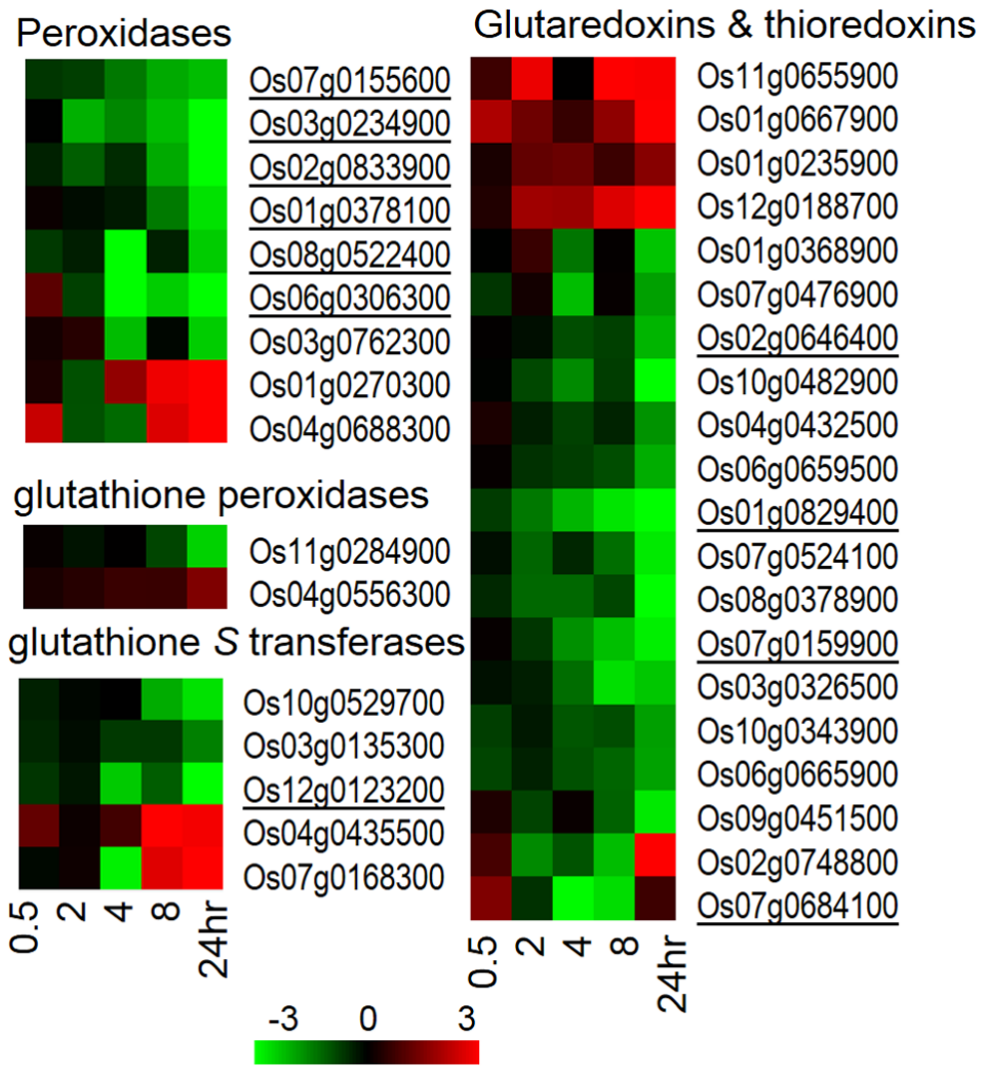
Clustering of genes associated with ROS scavenging. Hierarchical clustering of differentially expressed genes associated with ROS scavenging. JMOs are underlined.

Cold temperature causes oxidative stress leading to accumulation of reactive oxygen species (ROS), such as O_2_⋅**^_^**, H_2_O_2_, ^1^O_2_, HO_2_⋅**^_^**, OH**^.^**, ROOH, ROO**^.^**, and RO**^.^** [45]. ROS are highly reactive and induce deleterious effects on membranes that lead to ion leakage and also cause damage to proteins, DNA and lipids. Ultimately these effects result in cell death [45]. It has been shown previously that in the rice cv. Nipponbare, H_2_O_2_ levels increase within 1.5 hours of chilling stress (+10°C) [25]. To counteract the increased ROS levels, the cell produces scavengers such as peroxidases, catalases, ascorbate, glutathione, superoxide dismutase, glutaredoxins and thioredoxins that detoxify ROS [45]. In JM, 36 genes associated with ROS scavenging were cold induced. Among peroxidases, nine genes were differentially expressed, two up-regulated after eight hours and the remaining seven genes down-regulated after two to eight hours. Two glutathione peroxidases were also differentially expressed, one gene up- and one down-regulated after 24 hours, while glutathione S transferase was induced with two genes up-regulated at 8 hours and three down-regulated after four hours of cold stress. In addition, twenty genes belonging to glutaredoxins and thioredoxins were differentially expressed, with four genes up-regulated within the first two hours of cold stress and the remaining up-regulated during the 24 hour assay period. Out of the remaining 16 genes, one was up-regulated after 24 hours and the others were down-regulated (Figure 12, Table S5). Of the 36 genes, eleven genes were in the JMO set (underlined in Figure 12), one (Os01g0667900) was in the CCI set and four (Os03g0762300, Os11g0284900, Os12g0188700, Os08g0378900) were in the CIT set. Overall, this suggests differences in the ROS scavenging mechanisms in different rice cultivars.

### Cellular Components Play Distinct Roles under Cold Stress

**Figure 13:**
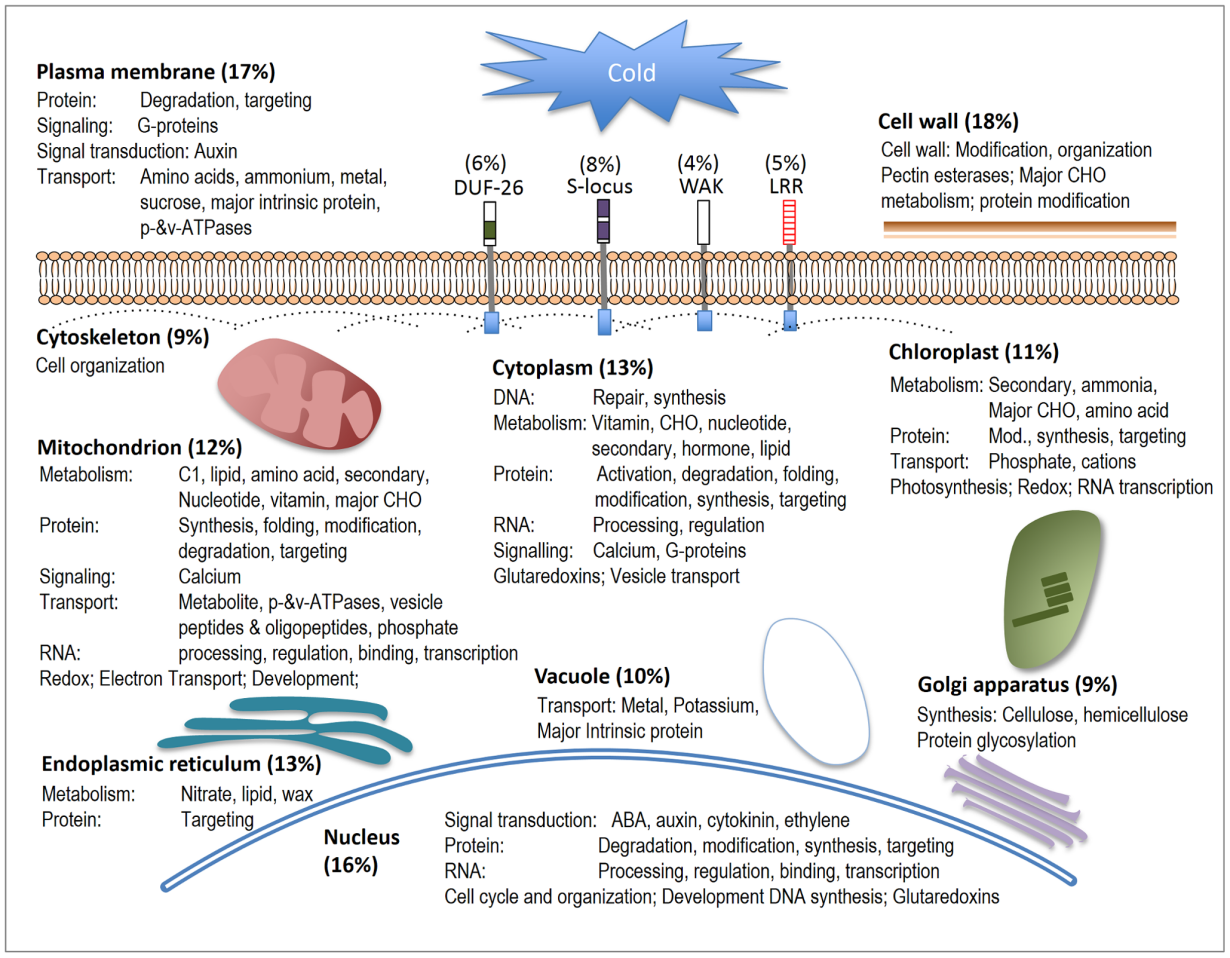
Overview of the role of cellular components under cold stress. Localization of genes in cellular components was identified from Gene Ontology. Molecular functions for the corresponding genes were identified from the MapMan annotations. More detailed annotations are in Table S1.

Organelles and sub-cellular components are highly affected by cold stress [46]. For example, in *Arabidopsis*, 184 nuclear proteins [47], 43 chloroplast proteins [48] and 38 plasma membrane proteins were identified as differentially expressed under cold stress [49]. Mitochondria, which are the prime sites for ROS production in abiotic stress, regulate ROS levels through their energy dissipating systems [50]. To evaluate if sub-cellular components play an active role during cold stress in JM, a functional annotation analysis was performed for DEGs encoding proteins predicted to be either organelle localized or associated to a cellular structure (hereon referred to as ‘orgLoc genes’) (Figure 13, Table S1).

The analysis showed that orgLoc genes from various cellular components were differentially expressed at a significant level during cold stress. The percentage of induced genes for each sub-cellular component or organelle was: cell wall (18%), plasma membrane (17%), nucleus (16%), cytoplasm (13%), endoplasmic reticulum (13%), mitochondria (12%), chloroplast (11%), vacuole (10%), Golgi apparatus (9%) and cytoskeleton (9%) (Figure 13). Moreover, several genes encoding plasma membrane receptors were also cold induced. A functional analysis of the orgLoc genes suggested distinct roles for different sub-cellular components and organelles during cold stress. For example, cold induced alterations in transport of metabolite process were found in the plasma membrane, mitochondria, chloroplast and vacuole, and signaling process in the plasma membrane, mitochondria and cytoplasm. Differential regulation in auxin signal transduction process could be seen in the plasma membrane, while, in the nucleus, auxin, ABA, cytokinin and ethylene signal transduction process were observed. Metabolism process of several compounds was also seen in the mitochondria, endoplasmic reticulum, cytoplasm, chloroplast and cell wall while differences in redox activity process were seen mainly in mitochondria and chloroplasts. Together this strongly suggests that the buildup of tolerance to low temperature stress involves many different components and that these components act synergistically.

### Identification of Cold Responsive Genes in QTLs Associated with Cold Stress

Previous studies have identified 22 different QTLs associated with cold stress in rice (hereon referred to as ‘cold QTLs’). To assess whether any of these QTLs overlap with stress response genes in JM, all 22 QTLs were downloaded from the Gramene database [51] and putative genes within the QTLs were identified and checked for homology to the JM DEGs.

**Figure 14:**
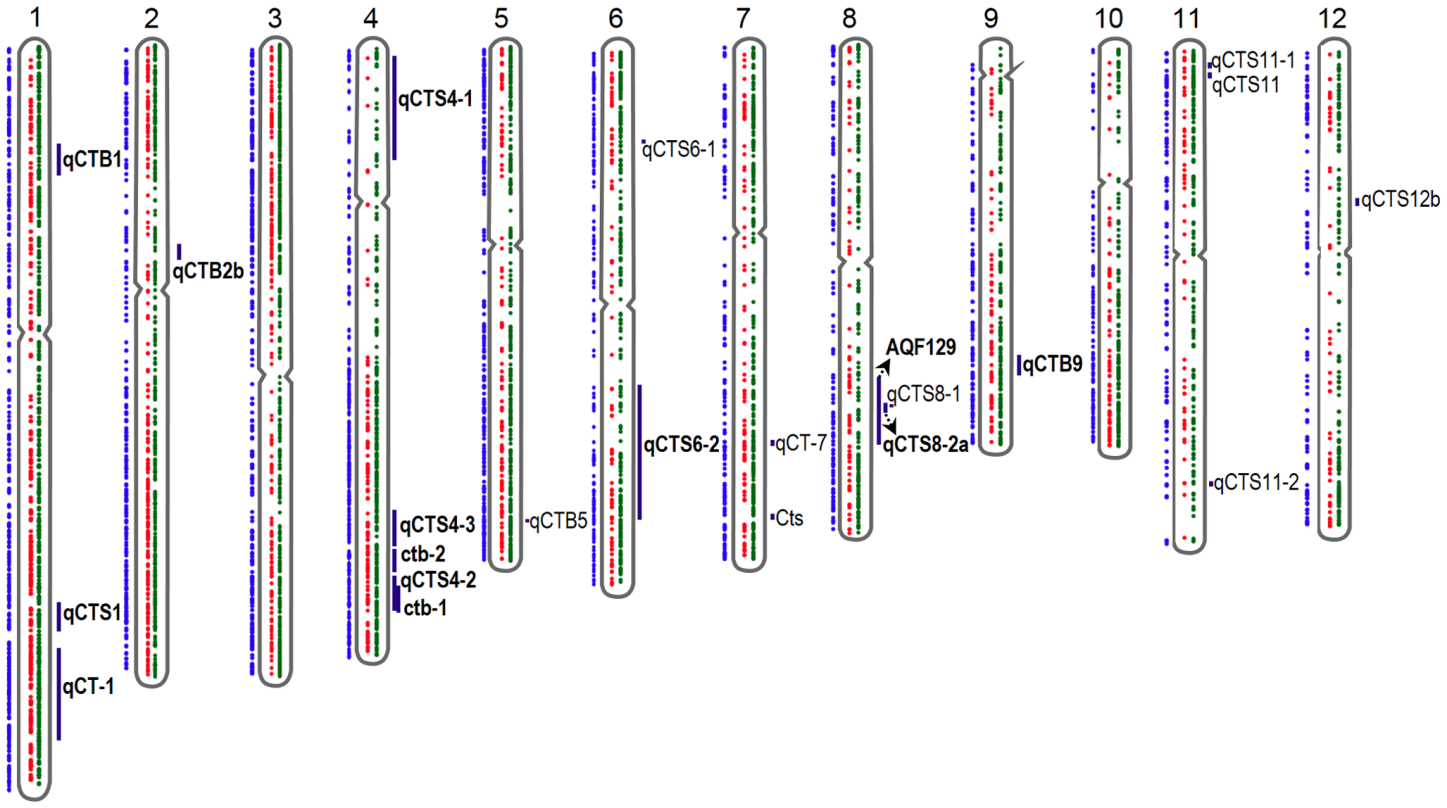
Localization of genes and QTLs on the chromosomes. Chromosomal localization of the 4,636 differentially expressed genes and 22 QTLs previously identified as associated with cold stress. Red dots indicate up-regulated genes, green indicate down-regulated genes and blue dots indicate JMO. QTLs are marked with vertical lines that cover their physical ranges. Physical locations of genes and QTLs were downloaded from the Gramene database, and the figure was generated with an R script.

This showed that 473 of the DEGs were present in 13 of the cold QTLs (Figure 14, Table S6) while no DEGs were found in the remaining 9 QTLs. MapMan annotations of the genes present in the 13 cold QTLs suggest distinct functional roles of these QTLs. The most common annotations were protein (62 genes), RNA (54 genes), signaling (27 genes), miscellaneous (22 genes) and transport (20 genes). All 13 QTLs also contained genes with yet unknown molecular functions. Of the 473 genes, 232 genes were found in the JMO set, while 40 and 12 genes were found in the CIT and CCI sets respectively (Table S7). The fact that several different QTLs contained DEGs with different functions suggests that cold tolerance is a multigenic trait. To increase cold tolerance by breeding, several different processes would, therefore, need to be successfully transferred to the same genome. As the cold QTLs were identified through genetic studies, it is highly likely that several of the cold induced genes located within the identified QTLs are critical for cold stress tolerance also in JM.

## Discussion

Low temperatures negatively affect both productivity and yield of rice grown in colder regions of the tropics. However, over the years, farmers and breeders have selected cultivars that are more chilling tolerant, resulting in rice cultivars that can even cope with cold stress to some extent. Since we were interested in the molecular mechanisms underlying this adaptation, we selected a chilling tolerant rice variety Jumli Marshi (JM) as a model and studied its transcriptional responses to cold stress. JM is a locally adapted endogenous variety that is routinely grown at up to 3,050 m in the Jumla district of Nepal. As a consequence of the high altitude, this variety is commonly exposed to temperatures down to or below 4°C during the early developmental stages in the growing season. JM is commonly used as a parental donor in various rice breeding programs worldwide for the development of new chilling tolerant rice cultivars. Chilling tolerant Nepalese rice cultivars Manjushree-2 and Khumal-8 were developed with JM as one of the parents [52]. JM was also successfully used as a parental donor for the development of upland chilling tolerant rice FOFIFA-172 in Madagascar [53].

JM has mostly been crossed with *indica* cultivars for the development of new chilling tolerant cultivars. Rice cultivar Khumal-8 is such an example where the parents are JM (spp. *japonica*) and IR36 (spp. *indica*). A study on the pedigrees of 28 Nepalese rice cultivars showed that 65.7% of the ancestors were of *indica* type and only 2.8% were *japonica* [54] again indicating that the parental types have mostly been indica Thus, in general a more detailed molecular comparison of *japonica* and *indica* sub-types is necessary for increased understanding of physiological, molecular and evolutionary aspects of chilling tolerance in rice. A few studies where chilling tolerant *japonica* rice cultivars have been compared to chilling sensitive *indica* ones exist [25], [27], but more comparisons with different cultivars are needed. Due to their superior agronomical characteristics, *indica* cultivars are most commonly used in Nepalese rice breeding programs, while, *japonica* cultivars are valued mainly for their chilling tolerance characteristics. Thus, comparative analysis of chilling sensitive *indica* and chilling tolerant *japonica* rice will facilitate in understanding of the chilling tolerance mechanisms and identify markers for breeding. In this work, a comparative transcriptome analysis was done between chilling tolerant upland Nepalese rice JM with a chilling tolerant japonica (LTH), and the two chilling sensitive *indica* rice cultivars PB1 and IR29. Adding chilling sensitive *japonica* rice to this analysis would have been of interest, but unfortunately there is no publicly available microarray data generated from chilling sensitive japonica rice.

Many of the cold induced genes derived in this study have previously been associated to cold stress in other systems [14], [16], [17], [23], [26], [27], [55], [56]. For example, *DREB1*s act as up-stream regulators during cold stress and are induced several folds within 30 minutes of cold treatment in several plant species [14], [16], [26], [27], [55], [57], [58], [59], [60], [61], [62]. Thus, many of the early cold induced genes seen in cold hardy plants are present in rice and the overall regulation of the upstream genetic regulators is also conserved. This indicates that some of the differences between chilling and freezing tolerant crops is not in the primary activation of cold acclimation pathways but may lie in the second wave of activation of downstream protection mechanisms or the existence of CBF-independent regulons in the chilling tolerant rice cultivars. However, the OsDREB1C regulon targets [38] are cold induced in both JM and IR64 (Figure S3b), which further strengthens the hypothesis that CBF-independent regulons play a critical role in chilling tolerance in rice.

In a previous study, Yun et al. [25] identified an early response putative regulatory network in *japonica* rice upon chilling stress at 10°C and suggested a strong integration of defense and growth related responses. Several well-known cold stress responsive TFs were differentially expressed including AP2, bZIP, MYB, WRKY, bHLH and NAC families. Functional annotations such as transport, signaling, defense, transcription and apoptosis were over-represented. In this work, although JM was cold stressed at 4°C, functional annotations common under chilling stress (10°C) were also found to be common under cold stress, indicating an overlap in regulatory mechanisms in chilling and cold stress. In a transcriptome profile comparison between LTH and IR29, Zhang et al. [27] identified 1,256 genes to be up-regulated only in LTH upon cold exposure. They also identified several functional annotations specific to either LTH or to IR29 or common to both. The results from this work further highlight genes, functional annotations and regulatory motifs that are specifically induced in JM.

Chlorophyll measurements suggest that IR64 plants suffer greatly within 48 hours of cold stress indicating that genes induced within the first 24 hours are critical for plant survival. This study identified 4,636 genes that were significantly differentially expressed within 24 hours upon cold stress in JM. Functional annotations of these genes showed that many of them are involved in gene regulation, post-translational modification, signaling, transport, development and metabolism. Comparative analysis with one other chilling tolerant rice and two chilling sensitive rice resulted in identification of 182 genes that were cold induced in all four rice cultivars (CCI genes). Also, 511 genes were induced in the two chilling tolerant cultivars (CIT genes). Promoter analysis of the CCI and CIT clusters gave further insight into similarities and differences among different cultivars. Motifs binding to CBF/DRE or ABRE TFs were over-represented in the promoters of CCI genes, thus suggesting that well-known cold induction pathways are conserved in rice cultivars differing in their chilling tolerance. Interestingly, ABRE elements were over-represented in the promoters of CIT genes, but CBF/DRE elements were not. This indicates that there are differences in the gene regulatory mechanisms in the chilling tolerant and sensitive lines. These differences could either be activation of parallel pathways that are independent of CBF regulation, or activation of pathways that are downstream of the CBF regulatory network. A further difference in the response of chilling tolerant and sensitive rice cultivars are the functional annotations of the DE genes. The top three annotations that were abundant in JM compared to the chilling sensitive cultivars were amino acid metabolism, RNA transcription and sugar transport.

Gene expression profiling under cold stress in *Arabidopsis* showed that the *AP2/ERF* family of TF genes was the most represented [63]. In *japonica* rice, it was shown that molecular responses triggered by oxidative signals are critical for survival under low temperature stress [25]. Under chilling stress, the chloroplast electron transport chain is over-reduced, resulting in increased production of ROS [64]. Less effective mitochondrial electron transport during cold stress can also lead to ROS formation [65], which can be detoxicated by different scavenging antioxidant enzymes produced by the plants. ROS scavengers, such as catalase [66], ascorbate peroxidase [67], glutathione reductase [68] and glutathione peroxidase [69] have been shown to play a role in eliminating free oxygen radicals during chilling stress. In rice, putative ROS mediated regulatory modules under chilling stress (+10°C) have been identified, and were suggested to be independent of DREB and ABA regulons [23]. In this work, genes involved in production of peroxidases, glutathione peroxidases, glutathione *S*-transferases, glutaredoxins and thioredoxins were significantly differentially expressed (Figure 12), indicating that ROS scavenging mechanisms are active in JM during cold stress (+4°C).

This study also shows that various cellular components play distinct roles during cold stress. Several orgLoc genes from nucleus, plasma membrane, endoplasmic reticulum and other components were differentially expressed in JM. The analysis reflects the physiological processes and downstream molecular mechanisms that possibly occur in the cellular components under cold stress. The plasma membrane acts as a primary barrier between the cytoplasm and the extracellular space and plays a critical role in cell integrity, signaling and transport [70]. In this work, plasma membrane orgLoc genes were involved in transport and the auxin signal transduction process. The cell wall provides cell integrity and support, and cell wall orgLoc genes were involved in the cell wall modification and organization process, reflecting changes occurring in the cell wall upon cold stress. The mitochondria and chloroplast are involved in metabolism of several compounds as well as energy generation from the electron transport chain. Again, several orgLoc genes with various functions differentially expressed in these cellular components indicate critical roles for these under cold stress. An overview of the processes taking place in each component during cold stress is shown in Figure 13.

Although efforts have been made to introduce increased chilling tolerance in rice by traditional breeding, the progress has been limited because chilling tolerance is a multigenic trait and involves several layers of protection to shield the plants from the stress. However, a number of QTLs that are statistically significantly associated with an increase in chilling tolerance have been identified through years of breeding. A list of such ‘cold QTLs’ from rice is summarized in Table S6. Since these QTLs are coupled to chilling tolerance, it is of interest to identify cold induced genes located within them. In a previous study, 445 cold induced genes from rice were located to 21 QTLs [27]. In this work, 473 of the cold induced JM genes were found to be located within 13 of the 22 previously identified cold QTLs (Figure 14, Table S6). Of these 473 genes, 232 genes were found in the JMO set, while 40 and 12 genes were found in the CIT and CCI sets respectively (Table S7). A more substantial increase in chilling tolerance is most likely only possible by a combined contribution from multiple QTLs. Thus, this and other similar studies on QTLs will facilitate the development of new markers and will further our understanding of chilling tolerance mechanisms in rice.

### Conclusion

Jumli Marshi is a highly adapted upland chilling tolerant Nepalese rice variety and is used extensively in breeding for new chilling tolerant rice cultivars both in Nepal and other countries. In this study, a global expression profiling was done to identify cold induced genes in JM. Comparative analysis resulted in identification of 2,101 genes DE only in chilling tolerant JM rice. From the comparison of JM and LTH, it can be concluded that there are significant differences in the way the two chilling tolerant japonica rice cultivars respond to cold, which further emphasizes the importance of including locally adapted landraces in scientific and breeding programs. In addition, in JM, 473 cold induced genes localized in previously described cold associated QTLs were identified and among these, 232 genes were DE only in JM. Further validation of these genes may lead to the development of new molecular markers for breeding of rice cultivars with increased chilling tolerance.

## Supporting Information

Figure S1 **MapMan annotations of genes induced by atleast 2 folds (log_2_) within 2 hours of cold exposure. Only annotations with two or more genes are shown.**
(TIF)

Figure S2 **Confirmation of expression levels of candidate genes by Quantitative- RT-PCR.** Relative expression levels at 24 h (in cold +4°C) compared to zero hour samples in Jumli Marshi and IR64 are shown. Expression levels were estimated by delta-delta CT method using the Biorad CFX manager software. Candidate genes and the primers are: Os05g0183100 (OsWRKY67) Forward: 5′CGCCGCTATCGACGCCAACT3′, Reverse 5′GTAGCGGTGGTCCTCCCGGT3′; Os03g0762300 (Similar to Peroxidase 51) Forward: 5′ATGCAGGCCACCATCCGCAC3′, Reverse: 5′GCACGTCGGTGCAGGAGACC3′; Os10g0510500 (SAUR family protein) Forward: 5′ GTGCGTGACGGTGAGGGTGG3′, Reverse 5′ AAGCGGGGGAGGTGGAGGTG3′.
(TIF)

Figure S3 **Venn diagram showing the number of genes from OsDREB1C regulon that are induced in JM upon cold stress.**
(TIF)

Table S1 **Expression profiles (log_2_) of differentially expressed genes.**
(XLSX)

Table S2 **Common and unique genes DE in different cultivars.**
(XLSX)

Table S3 **Over-represented words in CIT clusters.**
(DOCX)

Table S4 **MapMan annotations of differentially expressed genes.**
(XLSX)

Table S5 **Differentially expressed ROS scavengers.**
(XLSX)

Table S6 **Genes associated with cold QTLs.**
(XLSX)

Table S7 **JMO, CCI and CIT genes associated with cold QTLs.**
(XLSX)
