# Supplementary figures and images for: Global Expression Profiling of Low Temperature Induced Genes in the Chilling Tolerant Japonica Rice Jumli Marshi
Source: PLoS One. 2013 Dec 12;8(12):e81729. doi: 10.1371/journal.pone.0081729 (PMC3861252; doi:10.1371/journal.pone.0081729)

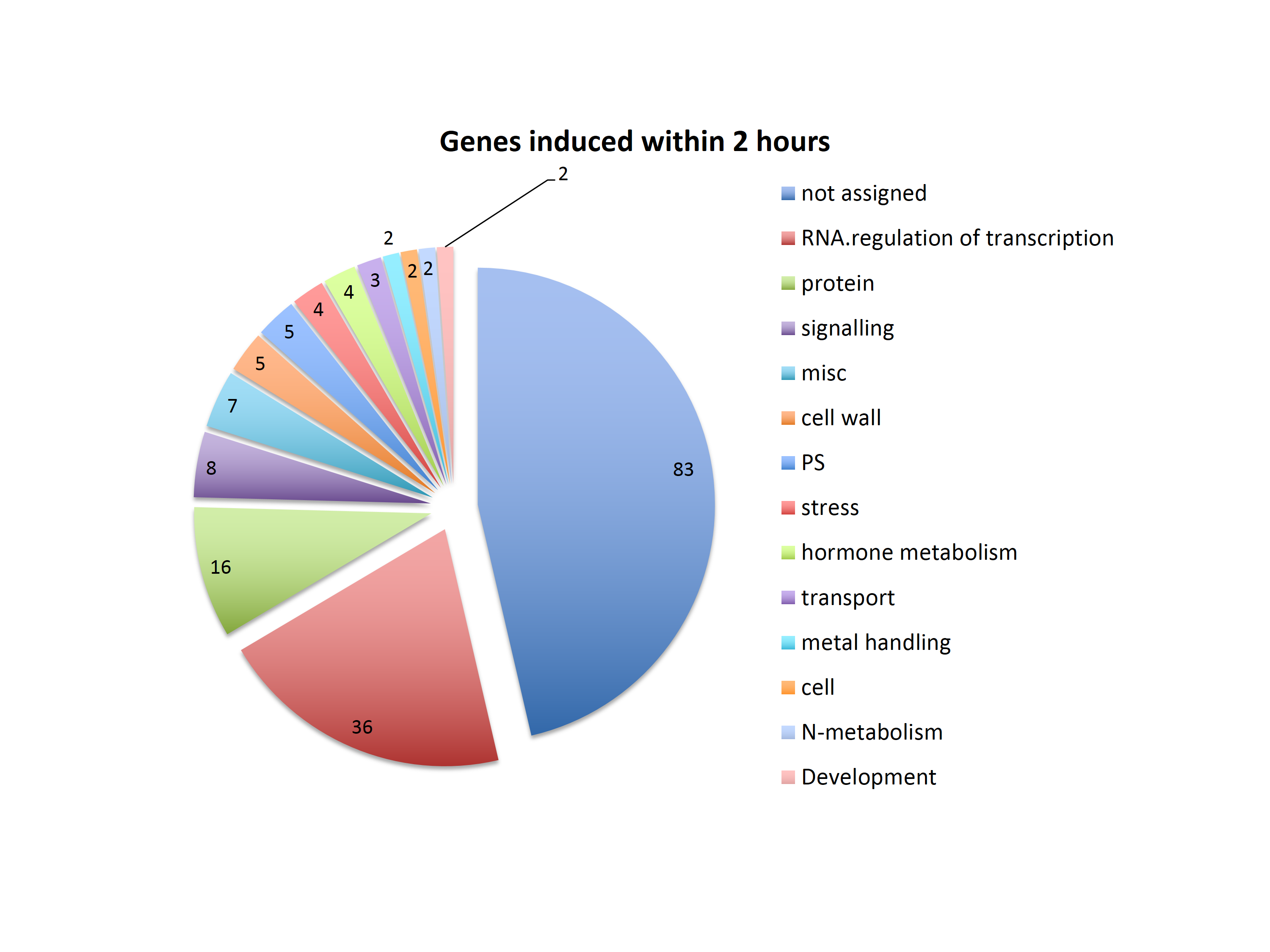

Supplement: Figure S1 — MapMan annotations of genes induced by atleast 2 folds (log2) within 2 hours of cold exposure. Only annotations with two or more genes are shown. (TIF) [file pone.0081729.s001.tif]

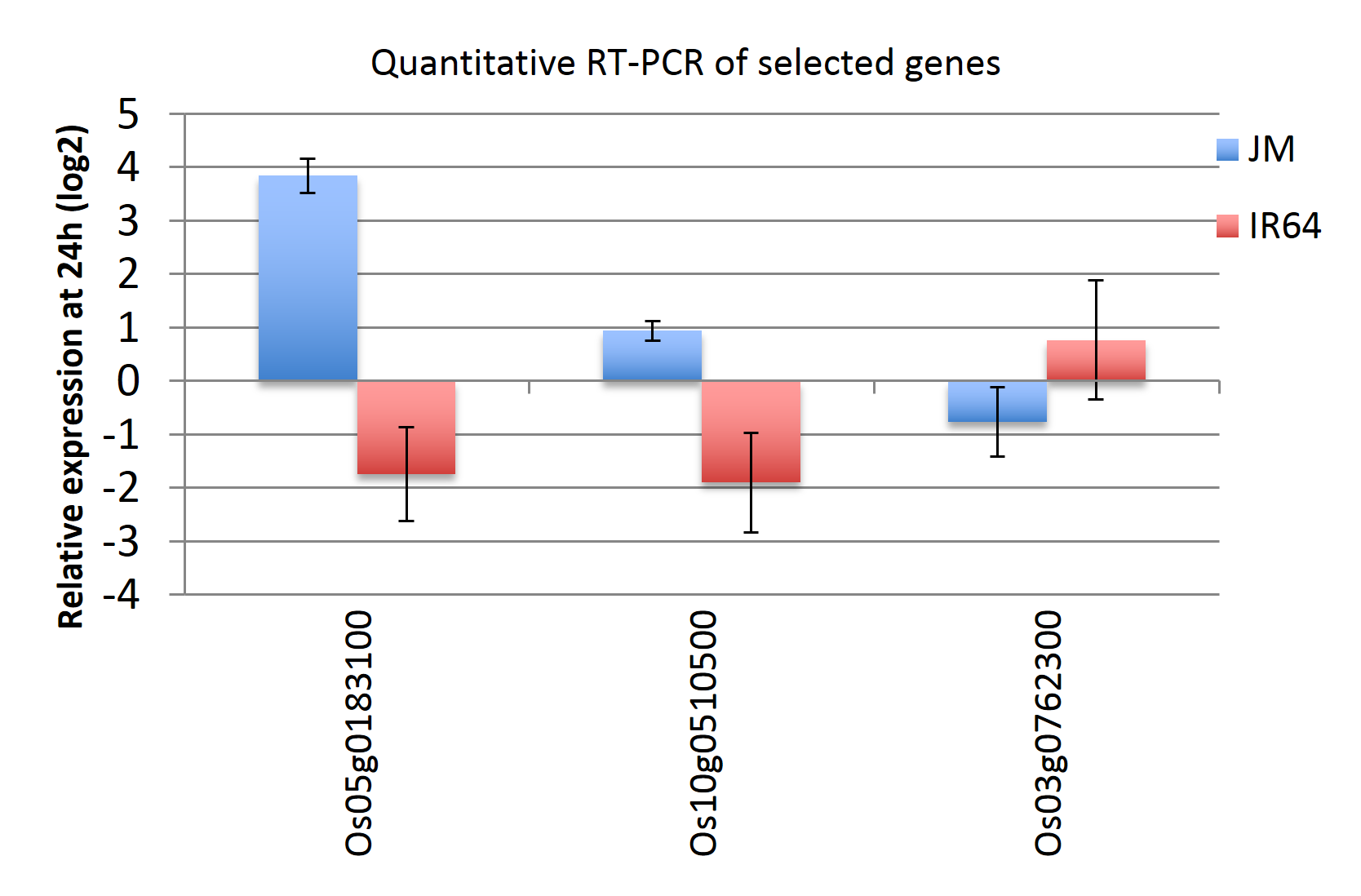

Supplement: Figure S2 — Confirmation of expression levels of candidate genes by Quantitative- RT-PCR. Relative expression levels at 24 h (in cold +4°C) compared to zero hour samples in Jumli Marshi and IR64 are shown. Expression levels were estimated by delta-delta CT method using the Biorad CFX manager software. Candidate genes and the primers are: Os05g0183100 (OsWRKY67) Forward: 5′CGCCGCTATCGACGCCAACT3′, Reverse 5′GTAGCGGTGGTCCTCCCGGT3′; Os03g0762300 (Similar to Peroxidase 51) Forward: 5′ATGCAGGCCACCATCCGCAC3′, Reverse: 5′GCACGTCGGTGCAGGAGACC3′; Os10g0510500 (SAUR family protein) Forward: 5′ GTGCGTGACGGTGAGGGTGG3′, Reverse 5′ AAGCGGGGGAGGTGGAGGTG3′. (TIF) [file pone.0081729.s002.tif]

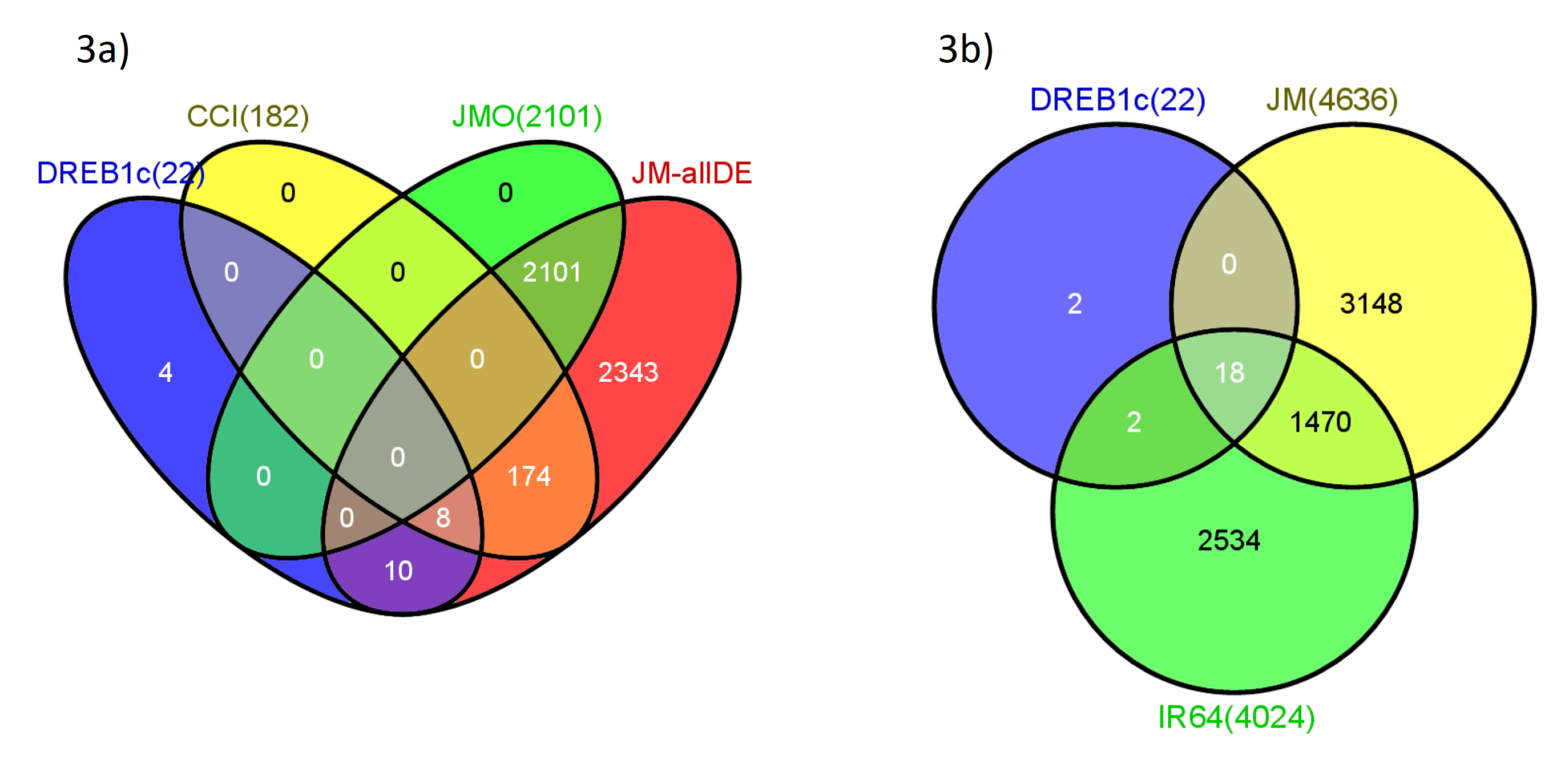

Supplement: Figure S3 — Venn diagram showing the number of genes from OsDREB1C regulon that are induced in JM upon cold stress. (TIF) [file pone.0081729.s003.tif]
