# Supplementary material for: Global Expression Profiling of Low Temperature Induced Genes in the Chilling Tolerant Japonica Rice Jumli Marshi
Source: PLoS One. 2013 Dec 12;8(12):e81729. doi: 10.1371/journal.pone.0081729 (PMC3861252; doi:10.1371/journal.pone.0081729)
Supplement: Table S3 — Over-represented words in CIT clusters. (DOCX) [file pone.0081729.s006.docx]

**##CIT Cluster 1**

Motif Total Hits Mean Hits Standard Deviation Hit Prob Hit Seq Hit P-value Mean P-value

### Cluster 0 ###

# Found in: HEXAT, SORLIP1AT, ABRELATERD1, ACGTATERD1, BOXIIPCCHS

CCACG 279 2.34454 2.44412 0.72268986 0.36938 4.47944e-06

CGTGGC 96 0.806723 1.21805 0.43697552 0.0191619 0.00015487

ACGTGGC 52 0.436975 1.02604 0.26890832 0.00119845 5.1966e-06

CCGCG 252 2.11765 2.86144 0.63865576 0.0145389 4.84273e-06

ACCGCGT 21 0.176471 0.38122 0.17647121 3.97057e-07 2.62283e-06

ACGCGGTC 9 0.0756303 0.264406 0.07563039 1.02817e-05 1.54157e-05

### Cluster 1 ###

# Found in: ABRELATERD1, ACGTATERD1, CACGTGMOTIF, IRO2OS, T/GBOXATPIN2

CACGC 231 1.94118 1.94588 0.72268986 0.142884 0.000297058

CACGCA 71 0.596639 0.955474 0.37815145 0.00881758 4.48251e-05

CAC 3524 29.6134 9.51788 1.0119 0.989464 0.00158898

CACG 844 7.09244 4.489 0.97479116 0.423354 8.55584e-09

ACACG 206 1.73109 1.35161 0.80672396 0.00240419 0.00111109

CGCG 454 3.81513 4.75265 0.80672396 0.0160103 2.17761e-08

ACGCG 189 1.58824 2.10007 0.59663971 0.0181504 1.35128e-06

CGCGTA 36 0.302521 0.783813 0.21008425 0.00998787 0.000287601

AACCGA 71 0.596639 0.872737 0.44537853 0.00039255 0.000240294

AACGAAG 25 0.210084 0.482883 0.18487422 0.000356943 0.000105283

ACGTG 251 2.10924 2.18394 0.76470691 0.00218981 1.2651e-07

ACGTGG 111 0.932773 1.59163 0.47058856 0.00193332 3.02175e-07

CACGTGG 49 0.411765 1.13324 0.19327723 0.0292617 4.54311e-06

AACTT 413 3.47059 3.06189 0.890756106 0.568491 0.00163307

ACGT 352 2.95798 2.20158 0.907563108 0.02048 2.21989e-06

AACGT 153 1.28571 1.33563 0.63865576 0.0238972 0.000806592

AACGTGCG 9 0.0756303 0.264406 0.07563039 1.34122e-05 1.85924e-05

CGC 2863 24.0588 19.3505 1.0119 0.543672 0.0010828

ACGC 612 5.14286 3.98376 0.957983114 0.12286 2.09824e-07

ACGCA 165 1.38655 1.46161 0.70588284 0.00714005 8.87728e-05

ACGCAC 60 0.504202 0.838906 0.33613440 0.00689963 8.72951e-05

ACGCACGT 11 0.092437 0.289642 0.09243711 4.58794e-06 7.9935e-06

### Cluster 2 ###

# Found in: POLLEN1LELAT52, GT1GMSCAM4, DOFCOREZM, TATABOX5

GAAA 1587 13.3361 6.29429 1.0119 0.924331 0.00167121

GAAAA 712 5.98319 3.53371 0.983193117 0.320167 2.27425e-06

AGAAAA 288 2.42017 1.83567 0.87395104 0.0116536 0.000120129

GAAAAA 323 2.71429 2.03805 0.907563108 0.000606334 1.35195e-07

GAAAAAA 144 1.21008 1.33394 0.64705977 0.00098705 8.85435e-05

AAA 9356 78.6218 30.3151 1.0119 0.981022 9.491e-30

AAAA 4102 34.4706 16.91 1.0119 0.72975 2.00519e-36

AAAAA 1900 15.9664 10.2514 1.0119 0.0065559 6.73846e-35

AAAAAA 967 8.12605 6.82993 0.932773111 5.81657e-06 3.16931e-24

AAAAAAA 548 4.60504 4.69976 0.80672396 3.84966e-05 2.01055e-18

AAAAAAAA 304 2.55462 3.25059 0.63025275 0.00112699 1.77399e-14

AAAAG 604 5.07563 3.0186 0.991597118 0.0409652 2.15752e-05

AAAAAG 258 2.16807 2.03052 0.81512697 0.0157401 4.64915e-06

AAAAAAG 132 1.10924 1.36456 0.59663971 0.00114353 3.89763e-06

AAAAAAGA 72 0.605042 0.98064 0.36134543 0.00439609 9.9079e-07

AAATA 806 6.77311 4.63896 0.966387115 0.179737 0.000182938

AAAATA 381 3.20168 2.94367 0.83193399 0.233923 1.70059e-05

AAAAATA 198 1.66387 1.83467 0.66386679 0.0109607 9.25921e-09

AAAAAATA 96 0.806723 1.07904 0.47058856 0.000820282 1.23084e-06

AAAAGAA 131 1.10084 1.57392 0.51260561 0.243374 2.54241e-05

AAAAAGA 122 1.02521 1.38699 0.52100862 0.0266616 2.10571e-06

AAAAAGAA 71 0.596639 1.06368 0.34453841 0.0172925 6.50454e-06

AAAT 2468 20.7395 10.0599 1.0119 0.696398 0.000269086

AAAAT 1047 8.79832 5.23174 1.0119 0.00827213 9.35287e-06

AAAAAT 456 3.83193 2.85892 0.907563108 0.0194717 2.45318e-07

AAAAATAA 82 0.689076 1.10574 0.38655546 0.026601 1.85222e-05

### Cluster 3 ###

# Found in: CURECORECR

TACTA 336 2.82353 2.39273 0.865546103 0.0378946 0.000466629

GTAGTA 114 0.957983 1.14051 0.56302567 0.000111664 3.74491e-06

CGTA 477 4.0084 2.83287 0.957983114 0.0689402 0.000814272

CGTAGCA 19 0.159664 0.429641 0.13445416 0.000433315 4.0084e-05

AATAC 295 2.47899 2.00356 0.865546103 0.0736527 0.00106672

GTA 3034 25.4958 8.13115 1.0119 0.964465 0.0024035

AGTA 1022 8.58824 4.77812 1.0119 0.241768 6.22224e-05

AGTAG 268 2.2521 1.86619 0.83193399 0.179669 0.000897227

CTACTA 96 0.806723 1.24534 0.47899257 0.0162253 0.000478899

CCTAC 177 1.48739 1.2221 0.75630390 0.0148977 0.0026265

GGTACGA 16 0.134454 0.408407 0.10924413 0.000910251 4.72696e-05

GACTCGA 19 0.159664 0.429641 0.13445416 0.000401802 5.01098e-05

CGACTCGA 11 0.092437 0.317331 0.084033610 8.87153e-06 2.6462e-06

### Cluster 4 ###

CAGCCTC 32 0.268908 0.545378 0.22689127 0.000342982 7.16153e-05

CAGCC 249 2.09244 1.71494 0.82352998 0.0254115 0.00172993

CAGCCA 86 0.722689 1.09179 0.46218555 0.00940209 0.00023615

AGGCTGG 25 0.210084 0.499982 0.17647121 0.000725111 9.08677e-05

AGGCTGGC 13 0.109244 0.337811 0.1008412 2.68817e-05 1.0766e-05

ACG 2626 22.0672 10.1995 1.0119 0.880173 1.01263e-08

AACG 627 5.26891 2.71534 1.0119 0.0263432 0.00159995

AACGC 130 1.09244 1.27016 0.59663971 0.0774158 0.00145189

GCAAACGC 11 0.092437 0.317331 0.084033610 1.26853e-05 3.19916e-06

### Cluster 5 ###

# Found in: SORLIP1AT, SORLIP2AT

CGCGA 183 1.53782 1.93504 0.59663971 0.0126459 1.81379e-06

CGCGAC 60 0.504202 0.896997 0.31932838 0.00372687 5.03815e-05

GGGCCAC 25 0.210084 0.54809 0.15126118 0.00574022 6.59299e-05

GCCAGCC 40 0.336134 0.651789 0.25210130 2.93709e-05 1.51121e-06

AGCCAGCC 16 0.134454 0.428489 0.10924413 0.000172307 1.2215e-05

### Cluster 6 ###

CCCC 1003 8.42857 8.52931 0.92437110 0.818132 1.26698e-09

CCCCA 240 2.01681 2.23036 0.73949688 0.498032 0.000290312

CCACAC 90 0.756303 0.978478 0.47899257 0.0121873 0.000130079

CCC 3234 27.1765 17.789 1.0119 0.968607 0.000415865

CCCA 918 7.71429 5.20204 0.991597118 0.622908 0.0028766

CCCAC 316 2.65546 3.1314 0.76470691 0.661261 5.21213e-05

CCCCAC 100 0.840336 1.65506 0.37815145 0.547167 0.000167566

CCCCCAC 36 0.302521 0.655354 0.21008425 0.0075175 0.000116023

CCAC 1153 9.68908 5.82771 1.0119 0.532154 2.9431e-07

ACCAC 282 2.36975 1.86009 0.848739101 0.195624 0.00287104

CCACCACC 39 0.327731 0.73461 0.21008425 0.000395068 5.76384e-06

CCCGC 206 1.73109 2.11715 0.62184974 0.164669 0.00151604

CCCCGC 80 0.672269 1.02169 0.38655546 0.00148328 6.43996e-06

CCCCCGC 31 0.260504 0.557036 0.21008425 0.000199557 0.000102124

CCACC 373 3.13445 3.13539 0.840336100 0.566736 0.00179993

CCCACC 127 1.06723 1.623 0.47899257 0.156061 1.30715e-06

CCCCACC 53 0.445378 0.932129 0.27731133 0.0043722 4.27126e-06

CCCCC 366 3.07563 5.11574 0.63865576 0.368732 4.31202e-12

CCCCCC 168 1.41176 3.73809 0.27731133 0.430078 2.20895e-14

CCCCCCC 100 0.840336 2.98169 0.1260515 0.219729 7.0463e-13

CCCCCCCC 79 0.663866 2.64504 0.06722698 0.216492 3.84792e-13

### Cluster 7 ###

# Found in: GT1GMSCAM4

TAAA 1970 16.5546 9.02443 0.991597118 0.894448 0.00106297

ATAAA 710 5.96639 3.95974 0.97479116 0.265529 0.00118407

ATGAA 429 3.60504 2.47413 0.915966109 0.535286 0.00129393

ATAA 1703 14.3109 7.88227 1.0119 0.43896 0.00293784

TATAA 568 4.77311 4.00092 0.915966109 0.322101 0.000728826

TGAAA 503 4.22689 4.06344 0.92437110 0.328271 4.95305e-05

TTGAAA 188 1.57983 3.1019 0.63025275 0.184941 2.4209e-06

GTTGAAA 49 0.411765 1.13324 0.27731133 0.00817479 7.69919e-05

TAAAA 857 7.20168 5.04364 0.97479116 0.237752 5.20834e-05

TAAAAA 395 3.31933 2.756 0.882353105 0.00471426 1.58505e-07

TTAAAA 314 2.63866 2.69681 0.77310992 0.105191 8.42062e-05

TGAAAAA 95 0.798319 1.01733 0.50420260 0.00240642 0.000132606

**#CIT Cluster 2**

Motif Total Hits Mean Hits Standard Deviation Hit Prob Hit Seq Hit P-value Mean P-value

### Cluster 0 ###

# Found in: GT1GMSCAM4, DOFCOREZM

CAAA 1961 16.0738 5.69033 1.0122 0.864053 3.02624e-11

ACAAA 583 4.77869 2.59417 0.991803121 0.0189676 2.57715e-05

CAAAA 719 5.89344 2.87102 0.983607120 0.243929 3.03993e-06

ACAAAA 238 1.95082 1.70271 0.79508297 0.0520894 6.08463e-05

CAAAAA 238 1.95082 1.24039 0.877049107 4.39943e-05 0.000605225

GAAA 1654 13.5574 5.04252 1.0122 0.922499 0.000132653

GAAAA 692 5.67213 2.96273 1.0122 0.0267129 0.000728993

GAAAAA 305 2.5 1.93437 0.844262103 0.0921202 0.000117834

AAC 4087 33.5 6.95448 1.0122 0.995618 7.95563e-06

AAAC 1574 12.9016 4.80677 1.0122 0.856591 1.13369e-05

AAAAC 586 4.80328 2.41796 0.983607120 0.0804603 0.0034974

AAAG 1429 11.7131 4.38043 1.0122 0.814639 0.000475578

AAAAG 601 4.92623 2.68318 0.983607120 0.117341 0.000357052

AAAAGA 251 2.05738 1.5749 0.852459104 0.00159029 2.54675e-05

AAT 6675 54.7131 16.3113 1.0122 0.953176 1.969e-19

AAAT 2787 22.8443 8.95253 1.0122 0.690075 4.526e-18

AAAAT 1115 9.13934 4.70318 0.983607120 0.135564 9.32524e-09

AAAAAT 454 3.72131 2.61227 0.92623113 0.00292406 4.49466e-06

AAAAATA 184 1.5082 1.51628 0.67213182 0.00600201 1.10351e-05

AAA 9914 81.2623 26.0488 1.0122 0.980548 1.67107e-49

AAAA 4119 33.7623 14.3596 1.0122 0.723977 4.97632e-30

AAAAA 1814 14.8689 8.14414 0.991803121 0.0361809 1.79269e-20

AAAAAA 904 7.40984 5.30659 0.942623115 7.94893e-07 2.36472e-13

AAAAAAA 507 4.15574 3.64179 0.852459104 9.33635e-08 1.71054e-10

AAAAAAAA 273 2.2377 2.34714 0.72131188 1.18295e-07 7.3638e-08

### Cluster 1 ###

# Found in: WBOXATNPR1, WRKY71OS

TGCAA 378 3.09836 2.38647 0.885246108 0.101145 0.000800219

TTCAA 434 3.55738 2.76705 0.959016117 0.0108431 0.00342722

AGCAAC 84 0.688525 0.713821 0.56557469 1.24586e-05 0.00264902

CCAA 1176 9.63934 3.75274 1.0122 0.816433 0.00947032

ACCAA 369 3.02459 1.85748 0.934426114 0.0332874 0.000153477

TCAA 1424 11.6721 4.56006 1.0122 0.714563 4.29585e-07

ATCAA 438 3.59016 2.20191 0.95082116 0.0568454 0.00275906

ATCAAA 181 1.48361 1.46676 0.72950889 0.013612 0.000268782

TCA 4365 35.7787 8.42957 1.0122 0.991375 8.16609e-11

ATCA 1317 10.7951 4.49761 1.0122 0.614531 1.70802e-06

ATCAT 418 3.42623 2.33617 0.893443109 0.520668 6.81528e-05

TGAA 1395 11.4344 4.91733 1.0122 0.65046 3.46253e-07

ATGAA 443 3.63115 2.29826 0.92623113 0.37362 0.000671712

ATGAAA 177 1.45082 1.42635 0.67213182 0.0610699 0.00012602

GTCAA 273 2.2377 1.80188 0.860656105 0.0505833 0.00179159

GTCAAA 142 1.16393 1.5169 0.5737770 0.00480263 3.45021e-08

### Cluster 2 ###

GTA 3082 25.2623 7.49951 1.0122 0.963586 0.00966365

AGTA 1046 8.57377 3.97929 0.991803121 0.574838 6.41048e-05

AGTAT 320 2.62295 1.76639 0.885246108 0.0151985 4.98366e-05

ACTA 1019 8.35246 3.90234 1.0122 0.183381 0.0110205

TACTA 356 2.91803 2.09053 0.92623113 8.18977e-05 3.31639e-05

AAGT 1113 9.12295 4.44193 0.991803121 0.894178 0.010025

AACTT 421 3.45082 2.63053 0.901639110 0.414636 0.00212827

AACCTC 74 0.606557 0.784662 0.4508255 0.000536845 5.90554e-05

CAA 5019 41.1393 8.78459 1.0122 0.991375 1.84084e-08

ACAA 1405 11.5164 4.70304 1.0122 0.774725 0.00997999

ACAT 1266 10.377 4.20836 1.0122 0.504587 0.00491022

AATGTC 68 0.557377 0.779335 0.40983650 0.00304024 0.00040605

### Cluster 3 ###

TTAA 881 7.22131 3.6429 0.983607120 0.215947 0.00231784

TTAAA 713 5.84426 3.73073 0.97541119 0.00114205 2.27707e-06

TCAAA 595 4.87705 3.09833 0.97541119 0.0159103 4.74636e-09

ATTTGA 196 1.60656 1.79049 0.71311587 0.0244206 0.000109227

TAAAA 889 7.28689 4.28967 0.983607120 0.0975875 9.03245e-06

TCAAAA 211 1.72951 1.38515 0.78688596 0.00168229 2.30886e-05

GTTTA 346 2.83607 2.02185 0.909836111 0.0432525 0.002518

TGAAA 527 4.31967 2.8234 0.942623115 0.113488 4.22144e-06

TGAACA 99 0.811475 0.994509 0.48360759 0.0514038 0.000432651

### Cluster 4 ###

# Found in: IBOX

TAAA 2170 17.7869 7.68992 1.0122 0.567973 5.43518e-11

ATAAA 741 6.07377 3.52315 0.991803121 0.0351662 0.000177315

ATAAG 317 2.59836 1.77256 0.918033112 0.00245319 0.00178854

ATAAT 572 4.68852 3.19344 0.959016117 0.0177521 0.000349875

ATAG 979 8.02459 4.00504 0.991803121 0.592157 0.00633863

ATAGA 346 2.83607 2.08571 0.877049107 0.197264 0.00111329

CTAAA 418 3.42623 2.26852 0.934426114 0.0431858 0.00184652

TATA 1029 8.43443 5.80558 0.983607120 0.207024 0.00790395

CTATA 347 2.84426 2.45958 0.819672100 0.396838 0.000286109

ATTA 1701 13.9426 6.74998 1.0122 0.306542 0.000368327

AATTA 671 5.5 3.63329 0.967213118 0.00604851 0.00227545

ACTTA 289 2.36885 1.78427 0.852459104 0.0330215 0.000387738

ACTTAT 117 0.959016 1.07427 0.5737770 0.00182348 4.61238e-05

### Cluster 5 ###

# Found in: GATABOX, IBOXCORE, TATABOX5

ATC 4044 33.1475 8.05175 1.0122 0.982704 0.000280177

AATC 1209 9.90984 3.60101 1.0122 0.703718 0.00128293

AATCA 419 3.43443 1.97105 0.959016117 0.0117683 0.00076529

TATCA 324 2.65574 1.76844 0.918033112 0.00244952 0.000748387

ATGA 1336 10.9508 4.47187 1.0122 0.688556 1.66799e-07

AATGA 383 3.13934 2.35503 0.885246108 0.481545 6.26218e-05

CATGA 299 2.45082 1.8647 0.868852106 0.0284332 0.00158674

GATGA 361 2.95902 2.11706 0.909836111 0.315457 0.00428345

GATA 976 8.0 3.38754 1.0122 0.204335 2.98091e-05

GATAA 323 2.64754 1.73641 0.893443109 0.0300104 8.05897e-05

TAA 5477 44.8934 15.2018 1.0122 0.967828 8.92845e-12

ATAA 1867 15.3033 6.86389 1.0122 0.429943 6.48006e-09

AATAA 696 5.70492 3.4181 0.959016117 0.185062 2.36362e-05

AAATAA 311 2.54918 1.91244 0.860656105 0.0047338 0.000178217

### Cluster 6 ###

# Found in: CAATBOX1

ACT 3529 28.9262 7.20561 1.0122 0.984865 0.00769118

AACT 1186 9.72131 4.02301 1.0122 0.581781 0.00312446

AAACT 460 3.77049 2.50503 0.918033112 0.496204 0.00230071

AACA 1356 11.1148 4.44308 1.0122 0.659037 0.0100409

AACAT 417 3.41803 2.261 0.934426114 0.047645 0.00012466

ATTG 1166 9.55738 3.95891 0.991803121 0.890011 0.00138737

CAATG 278 2.27869 1.63096 0.877049107 0.0109723 0.000146849

ATA 5956 48.8197 19.5876 1.0122 0.887042 2.09652e-09

AATA 1893 15.5164 7.3543 0.991803121 0.745345 2.25788e-05

AAATA 850 6.96721 4.05482 0.95082116 0.443049 4.37288e-06

AAAATA 400 3.27869 2.68042 0.860656105 0.0569623 1.37806e-06

ATG 4457 36.5328 8.70009 1.0122 0.984865 9.27207e-05

AATG 1214 9.95082 3.97709 1.0122 0.628075 4.95637e-06

AAATG 494 4.04918 2.37077 0.959016117 0.0556978 2.16939e-05

AAATGA 173 1.41803 1.49776 0.71311587 0.00498552 1.85496e-05

AATT 1178 9.65574 4.669 0.991803121 0.185453 8.87793e-06

AAATT 974 7.98361 5.01307 0.959016117 0.186492 2.35845e-09

AAATTA 266 2.18033 1.89916 0.81147599 0.0113259 0.000163827

### Cluster 7 ###

# Found in: SORLIP2AT

ATGGGC 95 0.778689 1.21137 0.45901656 0.0146475 0.000213302

GCCCA 319 2.61475 2.74144 0.73770590 0.577588 1.92276e-06

AGCCCA 95 0.778689 1.17007 0.4262352 0.0670411 8.18831e-05

GGCCCA 150 1.22951 1.67815 0.5245964 0.00614698 6.7837e-07

### Cluster 8 ###

ATTCA 384 3.14754 2.42484 0.885246108 0.31339 0.000249974

ATTTA 654 5.36066 3.1049 0.967213118 0.0707883 0.000150465

AATTTA 266 2.18033 1.87309 0.78688596 0.00641275 1.77718e-05

ATTTG 602 4.93443 2.65422 0.97541119 0.100116 7.72308e-08

AATTTG 218 1.78689 1.75195 0.73770590 0.0124158 8.90131e-06

ATTTTG 236 1.93443 1.80499 0.77868995 0.0544004 0.00029883

**##CIT Cluster 4**

Motif Total Hits Mean Hits Standard Deviation Hit Prob Hit Seq Hit P-value Mean P-value

### Cluster 0 ###

CAG 767 25.5667 9.99894 1.030 0.994644 0.00195648

CCTCAGG 9 0.3 0.458258 0.39 3.83267e-06 1.63043e-05

### Cluster 1 ###

# Found in: SORLIP2AT, ABFOS, HY5AT

GGCCC 99 3.3 3.36799 0.76666723 0.116046 2.86887e-07

AGGCCC 29 0.966667 1.32874 0.53333316 0.00949036 5.0775e-05

GCCCA 89 2.96667 3.2299 0.76666723 0.467468 0.000248232

GGCCCA 55 1.83333 2.14606 0.721 0.00117083 4.91651e-08

AGGCCCA 19 0.633333 0.982627 0.412 0.00069016 5.69104e-06

GATGCCA 14 0.466667 1.0562 0.2333337 0.0234789 6.65959e-06

### Cluster 2 ###

AAA 2426 80.8667 28.0532 1.030 0.995181 8.34362e-13

AAAA 1075 35.8333 15.5158 1.030 0.923647 1.49413e-14

AAAAA 521 17.3667 9.87753 1.030 0.28156 2.08756e-16

AAAAAA 286 9.53333 6.50504 0.927 0.0777995 2.46106e-15

AAAAAAA 176 5.86667 4.51467 0.927 0.00115867 1.2176e-14

AAAAAAAA 103 3.43333 3.25252 0.76666723 0.00165236 3.52339e-12

### Cluster 3 ###

AACT 326 10.8667 5.30869 0.96666729 0.992138 0.000414798

AAACT 142 4.73333 2.76807 0.93333328 0.505428 2.52494e-05

AAACTT 58 1.93333 1.74992 0.73333322 0.0824444 0.000114884

AGTTTG 43 1.43333 1.30852 0.721 0.020648 0.000106115

**##CIT Cluster 5**

Motif Total Hits Mean Hits Standard Deviation Hit Prob Hit Seq Hit P-value Mean P-value

### Cluster 0 ###

AATGT 79 3.7619 2.15815 0.95238120 0.138073 5.33682e-05

TACATACA 11 0.52381 1.70766 0.1904764 0.0322185 1.14141e-06

### Cluster 1 ###

GAA 806 38.381 9.70261 1.021 0.998867 0.00122765

AGAA 275 13.0952 5.57123 1.021 0.973338 0.0020634

TAA 939 44.7143 18.5296 1.021 0.994387 0.00395927

ATAA 334 15.9048 7.24999 1.021 0.864766 0.00110758

AAA 1771 84.3333 25.6317 1.021 0.996624 6.73662e-15

AAAA 781 37.1905 13.128 1.021 0.94592 5.51269e-14

AAAAA 363 17.2857 8.89164 1.021 0.411813 1.00897e-11

AAAAAA 187 8.90476 6.64691 0.95238120 0.0373012 1.7034e-08

AAAAAAA 106 5.04762 4.97112 0.80952417 0.0722169 2.0177e-06

### Cluster 2 ###

ATA 1099 52.3333 24.149 1.021 0.979579 1.08762e-06

AATA 347 16.5238 9.11628 1.021 0.845069 0.00214819

ATATA 190 9.04762 10.0687 0.95238120 0.283919 0.000217132

ATACATAC 11 0.52381 1.91781 0.1428573 0.0783635 2.28166e-07

### Cluster 3 ###

AAT 1163 55.381 22.9108 1.021 0.991779 1.88652e-05

AAAT 473 22.5238 11.7943 1.021 0.938143 0.000613464

AATT 218 10.381 6.84854 1.021 0.585738 0.00225221

AATTA 143 6.80952 5.36851 0.95238120 0.378342 0.000123495

### Cluster 4 ###

GAAA 312 14.8571 4.67298 1.021 0.98621 0.000922223

**##CIT Cluster 6**

Motif Total Hits Mean Hits Standard Deviation Hit Prob Hit Seq Hit P-value Mean P-value

### Cluster 0 ###

GTAA 181 7.86957 3.61524 1.023 0.700147 0.00351493

GTAAC 52 2.26087 2.60797 0.82608719 0.0313982 1.83442e-06

AGTTAC 20 0.869565 0.679152 0.69565216 2.33744e-05 0.000245941

GGTAACC 10 0.434783 2.03931 0.04347831 0.641165 6.98843e-07

CGGTAACC 9 0.391304 1.83538 0.04347831 0.220148 4.58515e-10

GATTA 72 3.13043 3.09704 0.95652222 0.0611041 0.00217726

GTTA 182 7.91304 3.47554 1.023 0.687115 0.000456506

GGTTA 51 2.21739 1.97719 0.91304321 0.0219176 0.00118039

CGGTTAC 10 0.434783 1.83744 0.08695652 0.254427 3.71288e-07

CGGTTACC 9 0.391304 1.83538 0.04347831 0.210373 1.44665e-10

TTAA 210 9.13043 4.70249 1.023 0.453722 2.89204e-06

ATTAA 143 6.21739 4.82707 0.95652222 0.25933 3.39671e-05

CTAA 228 9.91304 3.57422 1.023 0.844468 0.00128321

ATTAG 92 4.0 2.87417 1.023 0.0202719 7.86911e-06

GGTAAC 17 0.73913 2.02629 0.3478268 0.0557502 5.18596e-05

CGGTAAC 10 0.434783 1.83744 0.08695652 0.226763 2.75327e-07

GCGGTAAC 7 0.304348 1.42752 0.04347831 0.185762 2.76245e-08

GCGGTTAC 7 0.304348 1.42752 0.04347831 0.196827 2.32289e-08

### Cluster 1 ###

# Found in: ACGTATERD1

AAC 806 35.0435 5.87906 1.023 0.999172 0.000822086

AAAC 337 14.6522 3.80627 1.023 0.971239 1.64027e-05

AAACA 122 5.30435 2.13531 1.023 0.25885 0.00248234

AAACG 60 2.6087 1.24199 1.023 0.00105846 0.000319854

AAACGT 20 0.869565 0.946589 0.56521713 0.00238581 0.000215688

AATC 251 10.913 4.19019 1.023 0.935903 0.0024187

AATCA 100 4.34783 2.2767 1.023 0.0841158 0.000118007

AATCAT 41 1.78261 1.21428 0.82608719 0.00510491 3.60477e-05

TAAA 463 20.1304 8.28988 1.023 0.898845 2.37706e-08

ATTTA 149 6.47826 3.29518 1.023 0.19296 6.12004e-05

ATTA 390 16.9565 10.0628 1.023 0.800185 7.7843e-08

AATTA 163 7.08696 5.00359 1.023 0.094151 6.13179e-06

AATTAG 41 1.78261 2.06327 0.78260918 0.00756863 7.35464e-05

ACT 721 31.3478 6.63068 1.023 0.997129 0.000719293

ACTA 235 10.2174 3.70612 1.023 0.726313 3.14965e-05

ACTAA 82 3.56522 1.92977 1.023 0.0161243 0.000225395

ACTAAT 39 1.69565 1.54455 0.82608719 0.000414571 2.15779e-06

AATTAGT 21 0.913043 1.34853 0.52173912 0.000992143 4.78498e-06

### Cluster 2 ###

# Found in: DOFCOREZM, TAAAGSTKST1, ACGTATERD1

CAA 953 41.4348 7.59191 1.023 0.998368 0.00459151

CAAA 401 17.4348 4.83489 1.023 0.972828 3.85307e-06

CAAAC 92 4.0 1.88818 1.023 0.0965827 0.000209638

AAAG 291 12.6522 5.12122 1.023 0.962088 0.00310116

CTTTA 69 3.0 2.0 0.91304321 0.163849 0.00220579

CGTTTA 17 0.73913 0.895272 0.52173912 0.00206993 0.000245738

ACGTTTAA 7 0.304348 0.620994 0.2173915 0.000491581 1.04957e-05

### Cluster 3 ###

# Found in: GT1GMSCAM4

TCAAA 116 5.04348 3.36837 0.95652222 0.476166 0.00225181

TTAAA 171 7.43478 4.48985 1.023 0.0853153 1.8525e-07

AACA 281 12.2174 3.51341 1.023 0.9244 0.00507432

GAAAA 147 6.3913 3.44824 0.95652222 0.855272 0.00249519

GAAAAA 76 3.30435 2.59489 0.91304321 0.114849 3.5611e-05

AAA 2022 87.913 25.2292 1.023 0.996304 9.78478e-24

AAAA 836 36.3478 14.4902 1.023 0.940924 7.2842e-13

AAAAA 379 16.4783 8.88022 1.023 0.378447 8.70151e-10

AAAAAA 206 8.95652 5.48102 0.95652222 0.0245267 2.21286e-09

AAAAAAA 122 5.30435 4.31816 0.91304321 0.00290347 3.06119e-08

AAAAAAAA 71 3.08696 3.16108 0.65217415 0.0833756 3.46721e-07

### Cluster 4 ###

AGCTA 67 2.91304 2.55229 0.86956520 0.0983777 0.00157839

AAGCCAA 15 0.652174 1.23741 0.3043487 0.0582892 3.49813e-05

GTA 624 27.1304 6.31977 1.023 0.993031 0.0027312

AGTA 211 9.17391 2.98769 1.023 0.76002 0.00420138

TACTA 84 3.65217 2.52997 0.95652222 0.0380039 8.62562e-05

TAGGA 60 2.6087 2.53221 0.86956520 0.222683 0.00183949

AATCCTA 15 0.652174 1.08783 0.3478268 0.00930109 8.82974e-06

AATCCTAT 10 0.434783 0.970257 0.2173915 0.00310286 5.8678e-07

### Cluster 5 ###

# Found in: ROOTMOTIFTAPOX1

AAT 1380 60.0 16.224 1.023 0.991 1.3284e-13

AAAT 557 24.2174 8.59199 1.023 0.932455 1.54144e-07

AAAAT 225 9.78261 4.81805 1.023 0.395843 0.000268559

ATAT 260 11.3043 9.52997 1.023 0.526265 3.47028e-05

AATAT 149 6.47826 4.18973 1.023 0.080868 0.00035202

AATT 253 11.0 4.8544 1.023 0.556647 4.46199e-05

AAATT 209 9.08696 5.39656 1.023 0.212215 5.06011e-06

AAATTTA 36 1.56522 2.1023 0.60869614 0.0268741 1.366e-05

CTA 733 31.8696 7.32657 1.023 0.996716 7.16837e-05

ATAG 206 8.95652 4.7041 1.023 0.767324 0.00423518

TAA 1181 51.3478 18.5838 1.023 0.993854 3.31637e-14

ATAA 352 15.3043 6.5171 1.023 0.852882 0.00702762

ATAAA 160 6.95652 3.8615 0.95652222 0.751743 0.000587195

TATATA 67 2.91304 6.58663 0.47826111 0.728734 1.6144e-05

ATATATAT 49 2.13043 6.08851 0.1739134 0.550127 3.6753e-11

ATA 1233 53.6087 23.2412 1.023 0.977656 2.93371e-09

AATA 409 17.7826 5.90473 1.023 0.831629 3.4635e-06

AAATA 180 7.82609 3.35769 1.023 0.256155 0.000251002

AAATAT 76 3.30435 2.38537 0.86956520 0.133583 0.00017153

TATA 226 9.82609 8.34918 1.023 0.447968 0.000488817

ATATA 235 10.2174 15.088 0.91304321 0.493859 1.11394e-08

ATATAT 79 3.43478 6.8132 0.56521713 0.523681 5.91186e-08

ATATATA 112 4.86957 12.6811 0.3913049 0.528491 2.14813e-16

TATATATA 45 1.95652 5.86425 0.1739134 0.539545 2.20339e-09

### Cluster 6 ###

AGAGAGA 39 1.69565 2.8504 0.3913049 0.392569 5.45934e-05

AGAGAGAG 30 1.30435 2.57808 0.3043487 0.137007 8.31591e-06

GAGAGAGA 34 1.47826 2.46488 0.3913049 0.0473357 7.01224e-07

### Cluster 7 ###

AACCGCG 10 0.434783 2.03931 0.04347831 0.796022 1.46791e-05

AACCGCGC 6 0.26087 1.22359 0.04347831 0.336581 8.85375e-06

CGCGGTTA 6 0.26087 1.22359 0.04347831 0.320584 6.99399e-06
